# *All-trans-*retinoic acid modulates glycolysis via H19 and telomerase: the role of mir-let-7a in estrogen receptor-positive breast cancer cells

**DOI:** 10.1186/s12885-024-12379-3

**Published:** 2024-05-21

**Authors:** Rita El Habre, Rita Aoun, Roula Tahtouh, George Hilal

**Affiliations:** Cancer and Metabolism Laboratory, Faculty of Medicine, Saint-Joseph University, Beirut, Lebanon

**Keywords:** Breast cancer, Triple-negative breast cancer, Estrogen-positive breast cancer, *All-trans*-retinoic acid, Fulvestrant, H19, Telomerase, Let-7a microRNA, Pyruvate kinase M2, Lactate dehydrogenase A

## Abstract

**Background:**

Breast cancer (BC) is the most commonly diagnosed cancer in women. Treatment approaches that differ between estrogen-positive (ER+) and triple-negative BC cells (TNBCs) and may subsequently affect cancer biomarkers, such as H19 and telomerase, are an emanating delight in BC research. For instance, *all-trans*-Retinoic acid (ATRA) could represent a potent regulator of these oncogenes, regulating microRNAs, mostly let-7a microRNA (miR-let-7a), which targets the glycolysis pathway, mainly pyruvate kinase M2 (PKM2) and lactate dehydrogenase A (LDHA) enzymes. Here, we investigated the potential role of ATRA in H19, telomerase, miR-let-7a, and glycolytic enzymes modulation in ER + and TNBC cells.

**Methods:**

MCF-7 and MDA-MB-231 cells were treated with 5 µM ATRA and/or 100 nM fulvestrant. Then, ATRA-treated or control MCF-7 cells were transfected with either H19 or hTERT siRNA. Afterward, ATRA-treated or untreated MDA-MB-231 cells were transfected with estrogen receptor alpha ER(α) or beta ER(β) expression plasmids. RNA expression was evaluated by RT‒qPCR, and proteins were assessed by Western blot. PKM2 activity was measured using an NADH/LDH coupled enzymatic assay, and telomerase activity was evaluated with a quantitative telomeric repeat amplification protocol assay. Student’s t-test or one-way ANOVA was used to analyze data from replicates.

**Results:**

Our results showed that MCF-7 cells were more responsive to ATRA than MDA-MB-231 cells. In MCF-7 cells, ATRA and/or fulvestrant decreased ER(α), H19, telomerase, PKM2, and LDHA, whereas ER(β) and miR-let-7a increased. H19 or hTERT knockdown with or without ATRA treatment showed similar results to those obtained after ATRA treatment, and a potential interconnection between H19 and hTERT was found. However, in MDA-MB-231 cells, RNA expression of the aforementioned genes was modulated after ATRA and/or fulvestrant, with no significant effect on protein and activity levels. Overexpression of ER(α) or ER(β) in MDA-MB-231 cells induced telomerase activity, PKM2 and LDHA expression, in which ATRA treatment combined with plasmid transfection decreased glycolytic enzyme expression.

**Conclusions:**

To the best of our knowledge, our study is the first to elucidate a new potential interaction between the estrogen receptor and glycolytic enzymes in ER + BC cells through miR-let-7a.

**Supplementary Information:**

The online version contains supplementary material available at 10.1186/s12885-024-12379-3.

## Background

Cancer is one of the most prominent causes of mortality. Among females, breast cancer (BC) is the most commonly diagnosed cancer and the leading cause of cancer death [1]. BC can be categorized into the following groups: cells expressing estrogen receptor (ER+) or progesterone receptor (PR+), cells expressing human epidermal receptor 2 (HER2+), and triple-negative BC cells (TNBC) (ER−, PR−, HER2−). The treatment approaches of cells should be based on these molecular characteristics [2]. Estrogen receptor (ER) expression is the main indicator of potential responses to hormonal therapy, and approximately 70% of human BCs are hormone-dependent cells [3]. ER is produced by BC cells as two isoforms, the estrogen receptors alpha ER(α) and beta ER(β), which are the products of separate genes [4]. In fact, ER(α) overexpression is related to increased proliferation and metastasis [5], in addition to inhibited apoptosis of BC cells [6]. However, the role of ER(β) in BC remains elusive, as ER(β) may have a bi-faceted role in BC; it has both antiproliferative and pro-apoptotic activities, while a smaller number of studies suggest that ER(β) promotes the invasion and metastasis of BC [7]. ER is therefore a valuable target for BC therapy [8]. Fulvestrant is a pure antiestrogen with no known agonistic activity, contrasting tamoxifen. The steroidal agent fulvestrant prevents estradiol binding to ER(α) to a stronger extent than tamoxifen. It also has a distinct mode of action that causes severe receptor conformational changes, promoting receptor degradation and downregulation of ER protein level and depletion of ER transcriptional activation [9]. ERs provide a potential role in the regulation of long non-coding RNAs (lncRNAs), including H19 [10], and in the transcriptional regulation of human telomerase reverse transcriptase (hTERT) [11]. Telomerase is a nuclear reverse transcriptase enzyme that increases the length of telomeres; afterwards, it has recently emerged as an attractive target for cancer, as it is a crucial factor required for the tumor immortalization of cells [12]. In BC, the expression of hTERT is regulated by epigenetic, transcriptional, post-translational modification mechanisms and DNA variation [13]. Given the overexpression of hTERT in most BC cells, the detection of hTERT and its associated molecules are potential for enhancing early screening and prognostic evaluation of BC. Although still in its early stages, therapeutic approaches targeting hTERT and its regulatory molecules show promise as viable strategies for BC treatment [14]. Increased telomerase activity is observed in most malignant tumors [15]; therefore, different therapeutic approaches for telomerase, mainly specific inhibitors, have been developed to reduce tumorigenicity in BC [16]. Additionally, lncRNAs are involved in transcription, translational regulation, and cell development. They participate in the regulation of a variety of cell activities, such as cell differentiation, proliferation, invasion, and apoptosis, which may also occur through interacting with microRNAs (miRNAs) [17]. One of the lncRNAs with a crucial function in both embryonic development and tumorigenesis is the oncofetal lncRNA H19 [18]. . H19 lncRNA is highly expressed in a variety of human cancers and overexpressed in approximately 70% of BC [19]. H19 can play differential roles depending on the tissue type and developmental stage; it is an oncogene in BC and is highly expressed in cancer tissues compared with normal tissues [20]. In fact, the expression of H19 is higher in ER(α) positive cells than in ER(α) negative MDA-MB-231 cells, where overexpression of H19 is associated with increased proliferation, indicating that H19 favors BC development via different mechanisms [21]. H19 can regulate gene expression in BC at multiple levels, including epigenetic, transcriptional and posttranscriptional. The abnormal expression of H19 is closely associated with the tumorigenesis and progression of BC via different underlying molecular mechanisms. Indeed, a large number of clinical studies have suggested that H19 can serve as a potential biomarker for the diagnosis of BC [22]. Interestingly, H19 may interfere with the activity of the telomerase complex in cancer cells [23]. The impact of H19 on the metastatic abilities of human BC cells could be due to the sponging of miRNAs, such as regulating members of the let-7 miRNA family, which all play important roles in development, glucose metabolism, and cancer [24]. In addition, the overexpression of hTERT might enhance the invasiveness and metastatic ability of cancer cells through an interaction with miRNAs [25]. MiRNAs might play an important role in oncogenesis; therefore, abnormal miRNA expression can affect cell survival, tumor cell proliferation, apoptosis, metastasis, and invasion [26]. H19 interacts with miRNA pathways to regulate the expression of their targets. MicroRNA let-7 (MiR-let-7) is one of the earliest discovered miRNAs and has been reported to regulate self-renewal and tumorigenicity of BC cells; microRNA let-7a (miR-let-7a) is a new identified miRNA; it has been featured as a tumor suppressor in different human tumors by targeting genes implicated in tumors signaling pathways [27], which may open novel perspectives for clinical treatments against BC [28]. Decreased expression of miR-let-7a or impaired function of miR-let-7a could be associated with increased tumor metastasis [29]. Glycolysis is one of the signaling pathways regulated by miRNAs by targeting major transcription factors, enzymes, and oncogenes [30]. Therefore, the Warburg effect is a metabolic phenotype observed in tumor cells, in which the deregulation of miRNAs contributes to high glycolysis [31]. Pyruvate kinase (PK) and lactate dehydrogenase A (LDHA) are two crucial glycolytic enzymes that facilitate this process, conferring a growth advantage for tumor cells [32]. First, PK catalyzes the last step of glycolysis, the conversion of phosphoenolpyruvate (PEP) to pyruvate with concomitant ATP production [33]. Among the four isoforms of pyruvate kinase (PK) in mammals, L, R, M1, and M2, tumor cells predominantly express the M2 isoform PKM2 [34]. Second, LDHA is another crucial glycolytic enzyme that converts pyruvate to lactate and oxidizes the reduced form of nicotinamide adenine dinucleotide (NADH) to NAD + to sustain glycolysis [35]. Upregulation of pyruvate kinase M2 (PKM2) and lactate dehydrogenase A (LDHA) has been associated with tumorigenesis and reported in several malignancies, including BC cells; subsequently, these glycolytic enzymes pathways could be regulated by miRNAs, such as miR-let-7a [36, 37]. Retinoids are a family of signaling molecules that are natural and synthetic vitamin A derivatives [38], and they are known to inhibit the growth of hormone-dependent but not hormone-independent BC cells [39]. *All − trans −* Retinoic acid (ATRA), the prototype of retinoids, is involved in the regulation of multiple biological processes by activating specific genomic pathways or by influencing key signaling proteins [40]. ATRA has been widely investigated in preclinical and clinical trials to be used in the treatment of BC. It inhibits BC cell growth and prevents mammary carcinogenesis in animal models with the induction of cell apoptosis and cell-cycle arrest [41]. In addition, ATRA shows greater growth inhibition of BC cell for ER-positive than ER-negative cells, while triple negative BC cell such as MDA-MB-231 cell is poorly responsive to ATRA treatment [42]. Thereafter, ATRA could be considered a promising agent in the management of certain hematologic malignancies and solid tumors, including BC.

Subsequently, targeting H19, telomerase, and specific miRNAs, such as miR-let-7a, offers promising avenues for the treatment of BC by disrupting key processes involved in tumor progression and metastasis, which can enhance therapeutic efficacy, overcome resistance, and improve patient outcomes in a personalized manner.

The main objective of our study is to investigate a possible relationship among ATRA, H19, telomerase, and glucose metabolism in BC cells. This study focuses particularly on the modulation of the expression and activity of the enzyme PKM2 and the expression of LDHA in the glycolysis pathway, as well as the variation in the expression of miR-let-7a between MCF-7 (ER+) and MDA-MB-231 (ER-) BC cell lines.

## Methods

### Cell culture and treatment with reagents

The present study was performed on two BC cell lines, namely, MCF-7 (ATCC ®HTB-22™) and MDA-MB-231 (ATCC ®HTB-26™). The two cell lines were purchased from the American Type Culture Collection (ATCC, Manassas, Virginia, USA). Cells were cultured in 4.5 g/L Dulbecco’s modified Eagle’s medium (DMEM) (Sigma‒Aldrich, St. Louis, MO, USA) supplemented with 10% fetal bovine serum (FBS; Sigma‒Aldrich) and 1% penicillin/streptomycin (PS; Sigma‑Aldrich) according to the manufacturer’s protocol. All cells were cultured in a humidified atmosphere at 37 °C with 5% CO_2_.

Cells were seeded in 6‑well plates (2 × 10^5^ cells/well) or in 100 mm petri dishes (1.5 × 10^6^ cells/dish) for cell culture. At 80% confluence, cells were treated for 48 h with the following inhibitors: 5 µM *all − trans −* Retinoic acid (ATRA) inhibitor (Sigma‒Aldrich) and/or 100 nM fulvestrant (Sigma‒Aldrich). The negative control corresponded to non‑treated cells maintained in the same conditions as treated cells.

### Cytotoxicity assay

The cytotoxicity of ATRA and/or fulvestrant was evaluated using a WST-8 cell counting kit according to the manufacturer’s instructions (Sigma‒Aldrich, Germany). Briefly, 10^4^ cells per well were seeded in 96-well plates and incubated for 48 h in DMEM, 4.5 g/L (10% FBS, 1% PS). The medium was then removed and replaced with ATRA (1, 5, 10, 20 µM) and/or fulvestrant (0.1, 0.5, 1, 2, 5, 10, 20 µM). After 48 h, 10 µl of tetrazolium salt was added to each well. This assay uses tetrazolium salt, which is converted to the fluorescent product formazan by metabolically active cells. Fluorescence was monitored at 450 nm by a Multiskan Go ELISA reader.

### RNA extraction and quantitative reverse transcription polymerase chain reaction (RT‒qPCR)

Total cellular RNA from three independent experiments (biological replicates) was extracted using NucleoZol (MACHEREY–NAGEL; Bethlehem, PA, USA) reagent according to the manufacturer’s instructions. The RNA concentration and A260/A280 ratio were determined using a NanoDropTM 1000 spectrophotometer (Thermo Scientific). A total of 1000 ng of total RNA was reverse transcribed in a 20 µL total volume using the iScript cDNA Synthesis Kit (Bio-Rad, USA). The relative expression of the genes mentioned below was normalized to that of glyceraldehyde 3-phosphate dehydrogenase (GAPDH). Complementary DNA (cDNA) was amplified using a SYBR Green PCR Kit with a CFX Connect Real-Time PCR Detection System (Bio-Rad). The RNA levels were quantified using the 2^‑ΔΔCq^ method, and the treated samples were compared with their control. Primer sequences for GAPDH, H19, hTERT, PKM2, LDHA, ER (α) and ER (β) amplification are shown in Table 1 below.

**Table 1:** Primer sequences

Gene of interest	Forward	Reverse
GAPDH	CACCCATGGCAAATTCCATGGC	GCATTGCTGATGATCTTGAGGCT
H19	CCCACAACATGAAAGAAATGGTGC	CACCTTCCAGAGCCGATTCC
hTERT	CGGAAGAGTGTCTGGAGCAA	CTCCCACGACGTAGTCCATG
PKM2	CTGTGGACTTGCCTGCTGTG	TGCCTTGCGGATGAATGACG
LDHA	GATTCAGCCCGATTCCGTTAC	ACTCCATACAGGCACACTGG
ER(α)	CTGCGTCGCCTCTAACCT	TCCAGCTCGTTCCCTTGGAT
ER(β)	ATCGATAAAAACCGGCGCAAG	GAGCCACACTTCACCATTCC

### Quantitative RT‒PCR for detection of miRNAs

Total RNA was extracted as previously described. The expression of miR-let-7a was quantified by RT‒qPCR. Single strand RNA was first polyadenylated by poly(A) polymerase before reverse transcription into cDNA using qScript RT with a proprietary adapter oligo(dT) primer using the “miRCUY® LNA® RT Kit” (Qiagen) following the manufacturer’s protocol. The amplification step was carried out using the CFX Connect Real-Time PCR Detection System (Bio-Rad). U6 served as an internal control. The miRNA-specific primers are listed in Table 2 below.

**Table 2:** Sequences of miRNA primers

miRNA	Forward	Reverse
miR-let-7a	CGATTCAGTGAGGTAGTAGGTTGT	TATGGTTGTTCTGCTCTCTGTCTC
U6	ATTGGAACGATACAGAGAAGATT	GGAACGCTTCACGAATTTG

### ER(α) and ER(β) expression constructs

The ER(α) expression plasmid pEGFP-C1-ER alpha, ER(β) expression plasmid pCDNA3.1-nv5-ER beta and scramble vector Pbabe-neo were purchased from Addgene (Addgene plasmids #28,230, #22,770, and #1767, respectively). After being transformed using the heat shock technique, the *Escherichia coli* DH5α strain was spread using a sterile loop onto a prepared lysogeny broth (LB) agar plate containing kanamycin or ampicillin respectively, to isolate individual colonies of bacteria carrying the plasmids cited above and incubated overnight at 37$^ \circ {\rm{C}}$C. After 24 h, one colony was transferred into LB media with the corresponding antibiotic and incubated at 37$^ \circ {\rm{C}}$C for while shaking. After incubation, bacterial growth was characterized by a cloudy haze in the media. The plasmids were extracted and purified from the transformed and proliferated Escherichia coli DH5α using the GenElute HP Plasmid Maxiprep kit (Sigma‒Aldrich). MDA-MB-231 cells were then transfected using the traditional protocol with Attractene Transfection Reagent (Qiagen) following the manufacturer’s instructions. After 48 h of transfection, RNA and proteins were extracted as previously described.

### Western blot analysis

MCF-7 and MDA-MB 231 cells treated and/or transfected were harvested in PBS and lysed in 1% Triton lysis buffer supplemented in the presence of sodium orthovanadate, protease inhibitor cocktail, and phenylmethylsulfonyl fluoride (PMSF), all purchased from Sigma‒Aldrich, USA. The supernatant containing the protein was collected and concentrated by ultracentrifugation, and the protein concentration was measured using the BCA protein assay kit (Bio-Rad, USA). To evaluate the expression of PKM2 (58 kDa), ER(α) (59 kDa), ER(β) (59 kDa), and LDHA (38 kDa), proteins were separated on Tris-Glycine gradient polyacrylamide gels and transferred onto Immuno-Blot PVDF membranes (Bio-Rad). Membranes were incubated in blocking buffer, probed with antibodies specific for PKM2 (Cell Signaling, 1:1000 dilution), β-actin (Cell Signaling, 1:1000 dilution), LDHA (Cell Signaling, 1:1000 dilution), ER(α) (Cell Signaling, 1:1000 dilution), and ER(β) (Sigma, 1:500 dilution) at 4 °C overnight, washed, and then incubated with the appropriate peroxidase-conjugated secondary antibodies (Cell Signaling, 1:2000 dilution) at room temperature. Antibody binding was detected by incubation with enhanced chemiluminescence (ECL) reagents (Abcam) and exposure of the membrane in an ECL machine. The expression of the desired protein was compared to that of β-actin, which served as an internal control.

### SiRNA analysis

Small interfering RNAs (siRNAs) against H19 and hTERT, a positive control (all star cell death), and a negative control siRNA (all stars negative control) were purchased from Qiagen (Qiagen Inc., Valencia, CA, USA); transfection was performed according to the manufacturer’s recommendations. Briefly, 20 nM siRNA diluted with serum-free medium and Hi-perfect transfection reagent (Qiagen Inc.) were added to the wells (24-well plates and 6-well plates were used), with or without ATRA, and incubated at room temperature. After 15 min, MCF-7 cells were seeded and incubated for 72 h. The knockdown efficacy of H19 and hTERT, in addition to studying the effect of gene expression inhibition on ER(α), ER(β), H19, telomerase, miR-let-7a, PKM2, and LDHA in combination or not with ATRA treatment, was confirmed by RT‑qPCR.

### Assay of telomerase activity

Samples for telomerase activity assays were extracted from cells for use following standard methods. First, the cells were trypsinized, washed with PBS, centrifuged and resuspended in lysis buffer 3-[(3-cholamidopropyl)-dimethylammonio]-1-propane-sulfonate, 10 mM Tris pH 8.0 (Sigma‒Aldrich, USA). Second, the lysate was incubated on ice for 30 min and centrifuged at 13000 rpm at 4°C for 20 min. A BCA protein assay (Bio-Rad) was used to determine the protein concentration in the extracts. Heat-inactivated samples were used as negative controls. A real-time quantitative telomeric repeat amplification protocol (qTRAP) assay was performed. Briefly, reactions were carried out using a SYBR Green PCR Kit (Bio-Rad, USA). 1 µL cell lysate, telomerase primer TS (5’-AATCCGTCGAGCAGAGTT-3’) and reverse primer ACX.

(5ʹ-GCGCGGCTTACCCTTACCCTTACCCTAACC-3ʹ) were used. Samples were amplified for 40 cycles. Data analysis was performed with a CFX Connect Real-Time PCR Detection System (Bio-Rad) that incorporates the real-time PCR effectiveness that was calculated by successive dilutions of the most active sample.

### PKM2 activity assay

For activity, cells were lysed in buffer as described previously. Activity was measured using a NADH/lactate dehydrogenase (LDH) coupled assay. The decrease in OD at 340 nm due to the oxidation of NADH was monitored using a spectrophotometer. The reaction was started by adding 50 µg cell lysate to a mixture containing 50 mM Tris pH 7.5, 100 mM KCl, 5 mM MgCl2, 1.25 mM ADP, 0.5 mM PEP, 0.28 mM NADH and 8 units of LDH. Specific PKM2 activity was calculated per mg of cell lysate.

### Statistical analysis

One-way ANOVA followed by Tukey’s or Dunnett’s multiple comparisons test or an unpaired, two-tailed Student’s t-test was performed to analyze data from biological replicates using GraphPad Prism software. All experiments were repeated independently at least three times. A *p* value of ˂0.05 was considered statistically significant.

## Results

### ATRA and fulvestrant cytotoxicity on MCF-7 and MDA-MB-231 cells

**Fig. 1:**
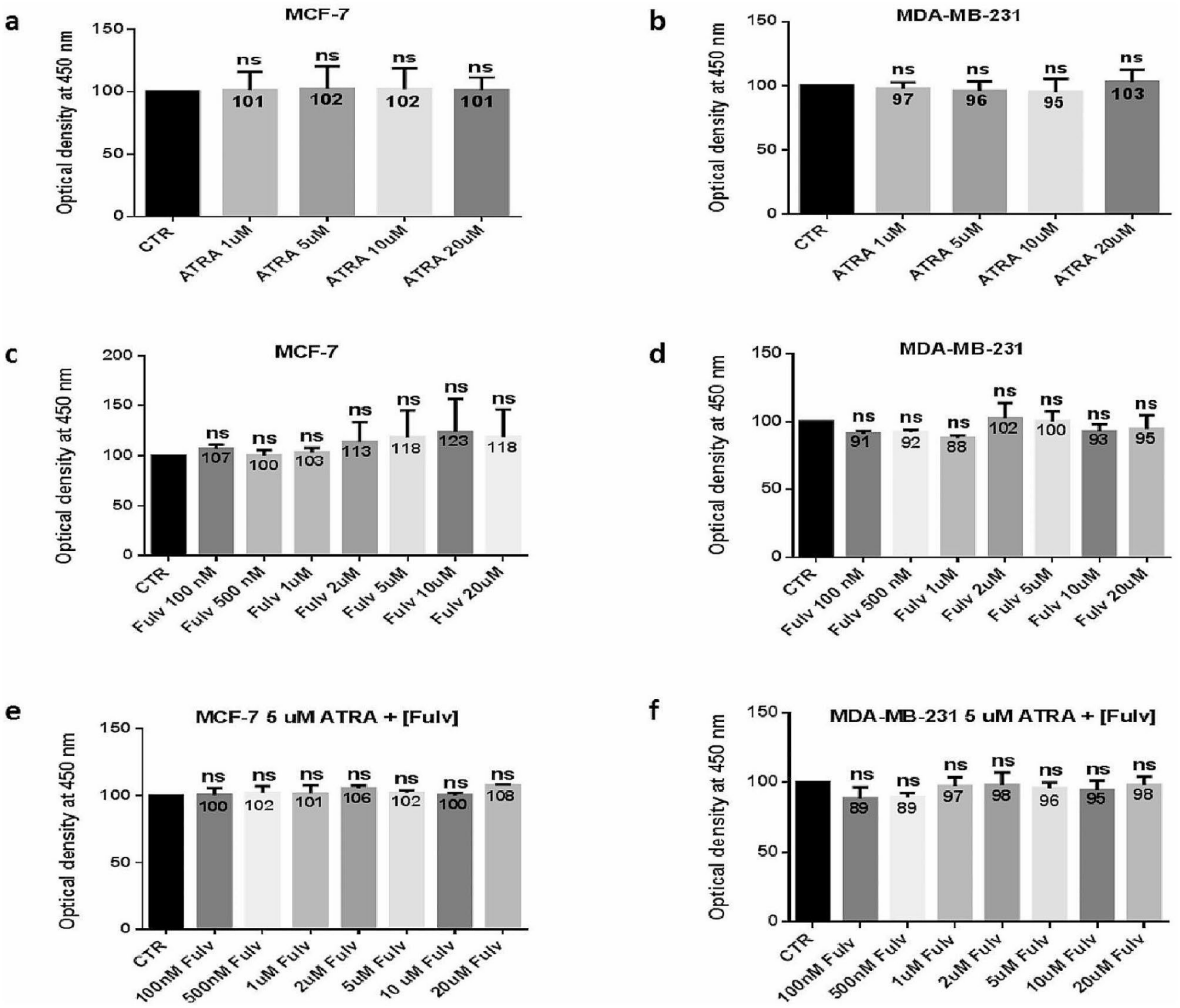
Effect of ATRA and/or fulvestrant on cell viability. MCF-7 and MDA-MB-231 cells were seeded at a density of 10^4^ cells per well in 96-well plates. Cell viability was calculated after 48 h of incubation and treatment and expressed as a percentage of control cells. This assay uses tetrazolium salt, which is converted to the fluorescent product formazan by metabolically active cells. Fluorescence was monitored at 450 nm by a Multiskan Go ELISA reader. The ATRA cytotoxicity effect on the cell lines was determined using different concentrations (1, 5, 10, and 20 µM) (**a, b**), and the fulvestrant cytotoxicity effect on the cell lines was determined using different concentrations (0.1, 0.5, 1, 2, 5, 10, and 20 µM) (**c, d**). After choosing and settling the appropriate concentration of ATRA (5 µM), the cytotoxicity of the ATRA and fulvestrant (various concentrations) combination toward the cells was evaluated as well **(e, f)**. Five replicates (*n* = 5) of each experimental condition were performed. Data are expressed as the mean ± SD of triplicates. ns; *p* > 0.05, as indicated

To indicate the concentrations that should be used for each inhibitor, a cytotoxicity test was performed, and the concentrations were chosen according to the highest concentration that has no toxic effect and therefore no effect on the viability of each cell line. After choosing and settling the appropriate concentration of ATRA (5 µM), the cytotoxicity of the ATRA and fulvestrant (various concentrations) combination toward the cells was evaluated as well. The cytotoxicity assay demonstrated that ATRA (Fig. 1. a-b) or fulvestrant (Fig. 1. c-d), as well as the combination of the two, was not cytotoxic toward MCF-7 (Fig. 1. e) and MDA-MB-231 (Fig. 1. f) cell lines at any concentration tested compared to the control after 48 h of treatment.

### Effect of ATRA treatment on ER isoforms, H19 and hTERT RNA expression in MCF-7 and MDA-MB-231 cells

**Fig. 2:**
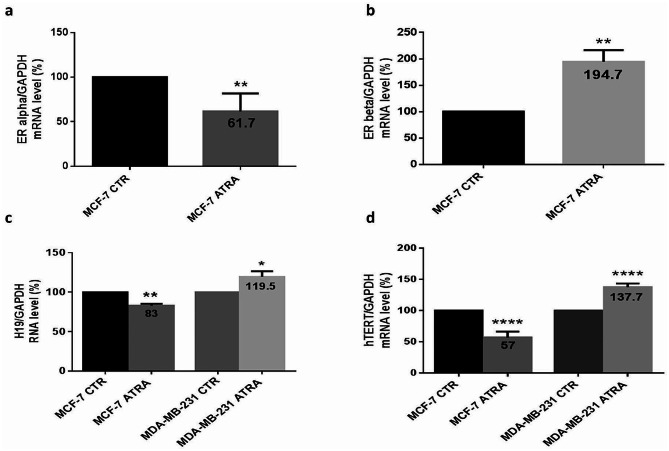
Effect of ATRA on the estrogen receptor isoforms H19 and hTERT in MCF-7 and MDA-MB-231 cells. MCF-7 and MDA-MB-231 cells were seeded at a density of 1.5 × 10^6^ cells per dish and treated with 5 µM ATRA for 48 h. RNA was extracted from both cell lines, and the quantification of the expression of ER(α) (**a**), ER(β) (**b**), H19 (**c**), and hTERT (**d**) was performed by qPCR using SYBR green mix. Data are the mean ± SD from three independent experiments with differences calculated using the delta-delta Ct method relative to the expression of the reference gene GAPDH. Each value represents the mean of three assays. Data are expressed as the mean ± SD of triplicates. *p < 0.05, **p < 0.01, ***p < 0.001, ****p < 0.0001 as indicated

Considering ATRA as a promising agent for BC cell treatment that could be involved in the regulation of multiple biological processes by influencing specific genomic pathways and with the aim of differentiating the effects of ATRA on MCF-7 and MDA-MB-231 cells, both cell lines were treated with 5 µM ATRA for 48 h in 4.5 g/L high glucose DMEM. RNA expression of ER(α) and ER(β) was evaluated in ER-positive cells; however, H19 and hTERT were evaluated in both cell lines. RNA expression was quantified using the primer sequences mentioned in Table 1. As shown in Fig. 2. We found that in MCF-7 cells, ATRA significantly decreased ER(α) (38.3%) (*p* = 0.0088) (Fig. 2. a), H19 (17%) (*p* = 0.0022) (Fig. 2. c), and hTERT (43%) (*p* < 0.0001) (Fig. 2. d), whereas ATRA significantly increased ER(β) (94.7%) (*p* = 0.0017) (Fig. 2. b). However, in MDA-MB-231 cells, ATRA significantly increased H19 (19.5%) (*p* = 0.03) (Fig. 2. c) and hTERT (37.7%) (*p* < 0.0001) expression (Fig. 2. d).

### Effect of ATRA and/or fulvestrant on ER(α), ER(β), H19, and telomerase in MCF-7 cells, as well as on H19 and telomerase in MDA-MB-231 cells

**Fig. 3:**
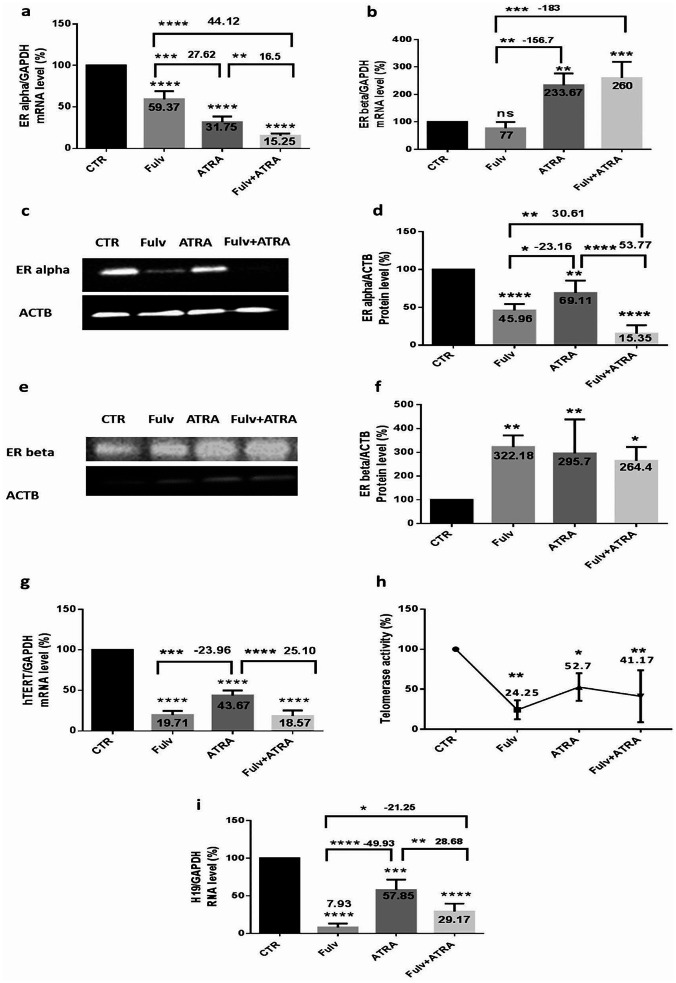
ATRA and/or fulvestrant modulates ER(α), ER(β), H19, and telomerase in MCF-7 cells. MCF-7 cells were seeded at a density of 1.5 × 10^6^ cells per dish and treated with 5 µM ATRA and/or 100 nM fulvestrant for 48 h. RNA was extracted from cells, and the quantification of the expression of ER(α) (**a**), ER(β) (**b**), hTERT (**g**), and H19 **(i)** was performed by RT‒qPCR using SYBR green mix. Data are the mean ± SD from three independent experiments with differences calculated using the delta-delta Ct method relative to the expression of the reference gene GAPDH. After treatment, the cells were lysed, and 50 µg of extracted proteins was analyzed using Western blotting with β-actin as an internal control for MCF-7 cells. Representative Western blot showing the change in protein levels of ER(α) (**c**) and ER(β)(**e**) compared to β-actin. The bar graph shows the quantified protein levels (**d-f**). Three independent experiments were carried out, and a representative image is shown. Full-length blots are presented in Supplementary Figs. 1 and 2. After treatment, telomerase activity was detected using a qTRAP assay (**h**, **i**). Each value represents the mean of three assays. Data are expressed as the mean ± SD of triplicates. ns; *p* > 0.05, *p < 0.05, **p < 0.01, ***p < 0.001, ****p < 0.0001 as indicated

**Fig. 4:**
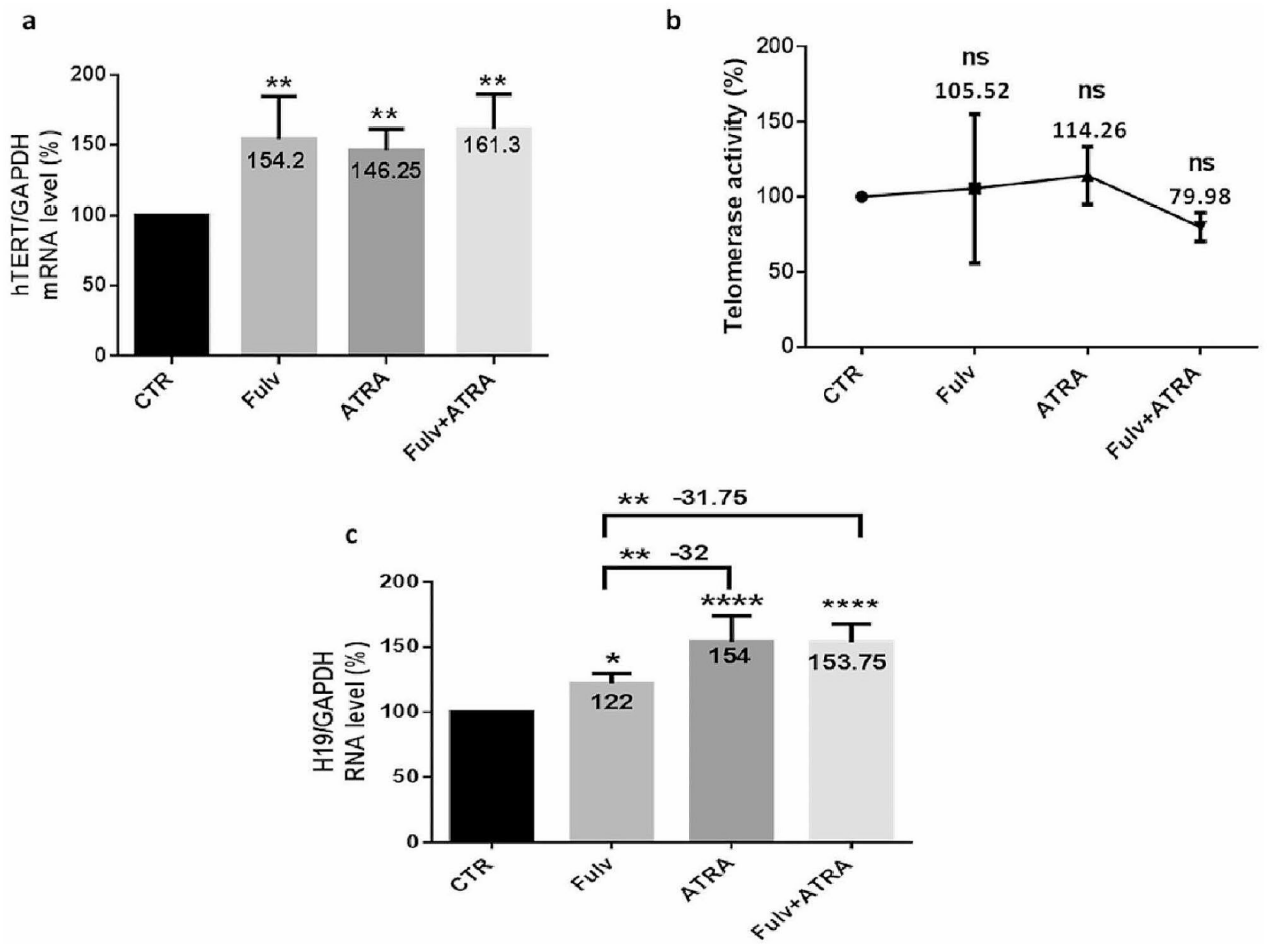
Effect of ATRA and/or fulvestrant on H19 and telomerase in MDA-MB-231 cells

Knowing that ER is a valuable target for BC therapy, to evaluate the effect of ATRA on MCF-7 ER-positive cell lines, which act as hormone-dependent cells, and to highlight the importance of ER isoforms in modulating H19 and hTERT expression, cells were treated with 5 µM ATRA inhibitor and/or with 100 nM fulvestrant for 48 h. RNA expression of ER(α), ER(β), H19, and hTERT was evaluated by Q-PCR. ER(α) and ER(β) proteins were quantified using Western blot analysis, and telomerase activity was evaluated with a qTRAP assay. The results shown in Fig. 3 reveal the variation in MCF-7 cells treated. ER(α) mRNA (Fig. 3. a) and protein expression (Fig. 3. c-d) decreased significantly after all the treatments; mainly, the inhibitor combination showed a very strong significant decrease in ER(α) (84.75%) (*p* < 0.0001). Our results showed a significant increase in ER(β) mRNA (Fig. 3. b) and protein (Fig. 3. e-f) expression; note that the treatment combination significantly increased ER(β) mRNA (160%) (*p* = 0.0001) and protein expression (164.4%) (*p* < 0.05). hTERT, which acts as a crucial factor required for the tumor immortalization of cells, was subjected to treatment combination and showed a significant decrease in mRNA and activity, with expression decreasing significantly at the mRNA level (Fig. 3. g) by 81.43% (*p* < 0.0001) and activity (Fig. 3. h) by 58.83% (*p* = 0.002). H19, an oncogene in BC development, showed a highly significant decrease after treatment combination (Fig. 3. i) (70.83%) (*p* < 0.0001). However, to differentiate the variations mentioned above in TNBC cells, MDA-MB-231 cells were subjected to the same treatment conditions as MCF-7 cells. In MDA-MB-231 treated cells, we found that hTERT mRNA expression increased significantly, mostly after inhibitor combination (Fig. 4. a) (61.3%) (*p* = 0.001), while Fig. 4. b shows no significant variation in telomerase activity after treatment. Although, H19 RNA expression increased significantly mainly after the combination treatment (Fig. 4. c) (53.75%) (*p* < 0.0001).

MDA-MB-231 cells were seeded at a density of 1.5 × 10^6^ cells per dish and treated with 5 µM ATRA and/or 100 nM fulvestrant for 48 h. RNA was extracted from cells, and the quantification of the expression of hTERT **(a)** and H19 **(c)** was performed by RT‒qPCR using SYBR green mix. Data are the mean ± SD from three independent experiments with differences calculated using the delta-delta Ct method relative to the expression of the reference gene GAPDH. After treatment, telomerase activity was detected using a qTRAP assay **(b)**. Each value represents the mean of three assays. Data are expressed as the mean ± SD of triplicates. ns; *p* > 0.05, *p < 0.05, **p < 0.01, ***p < 0.001, ****p < 0.0001 as indicated.

### Glycolysis modulation after ATRA and/or fulvestrant treatment of MCF-7 and MDA-MB-231 cells

**Fig. 5:**
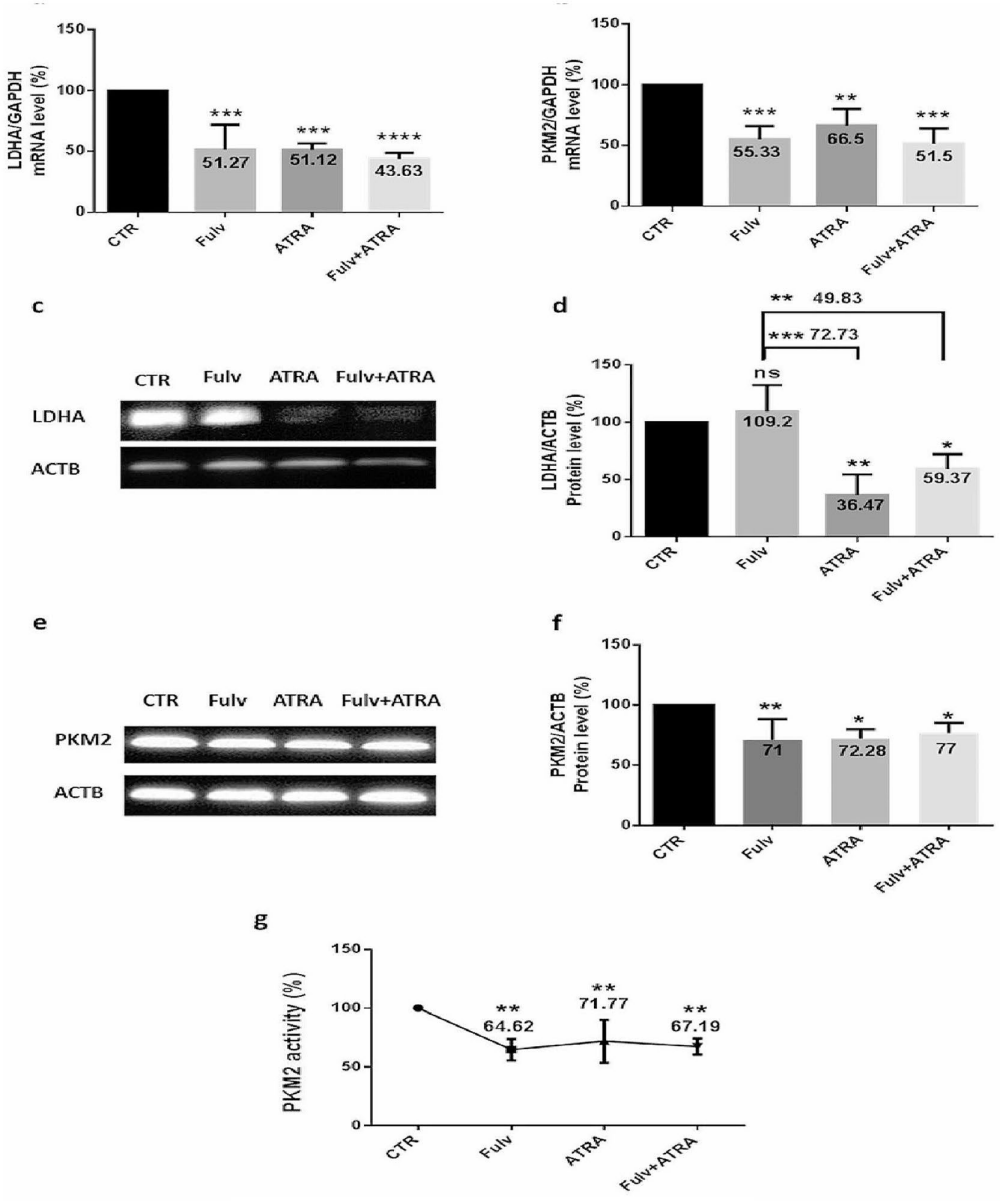
ATRA and/or fulvestrant modulates glycolytic enzymes in MCF-7 cells. MCF-7 cells were seeded at a density of 1.5 × 10^6^ cells per dish and treated with 5 µM ATRA and/or 100 nM fulvestrant for 48 h. Effectively, mRNA was extracted from cells, and the quantification of the expression of LDHA (**a**) and PKM2 (**b**) was performed by RT‒qPCR using SYBR green mix. Data are the mean ± SD from three independent experiments with differences calculated using the delta-delta Ct method relative to the expression of the reference gene GAPDH. After treatment, cells were lysed, and extracted proteins were analyzed using Western blotting with β-actin as an internal control for MCF-7 cells. Representative Western blot showing the change in protein levels of LDHA (**c**) and PKM2 (**e**) compared to the loading control. Full-length blots are presented in Supplementary Figs. 3 and 4. The quantitative analysis of the intensity of the bands is shown in the bar graph (**d-f**). After treatment, PKM2 activity was examined using an NADH/lactate dehydrogenase (LDH) coupled assay (**g**), where a decrease in optical density (OD) at 340 nm indicates a decrease in cell PKM2 activity. Each value represents the mean of three assays. Data are expressed as the mean ± SD of triplicates. ns; *p* > 0.05, *p < 0.05, **p < 0.01, ***p < 0.001, ****p < 0.0001 as indicated

**Fig. 6:**
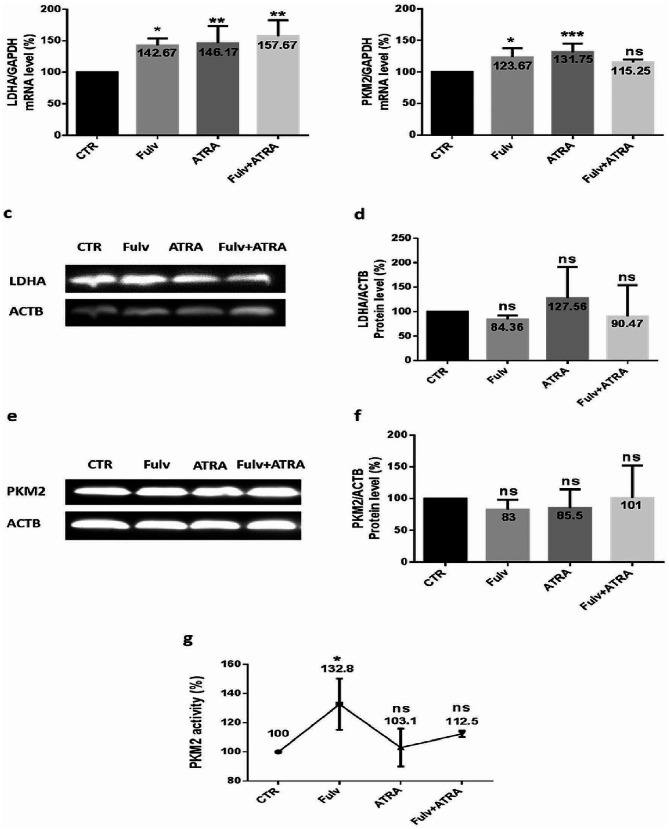
Effect of ATRA and/or fulvestrant on glycolytic enzymes in MDA-MB-231 cells. MDA-MB-231 cells were seeded at a density of 1.5 × 10^6^ cells per dish and treated with 5 µM ATRA and/or 100 nM fulvestrant for 48 h. First, mRNA was quantified, and the expression of LDHA (**a**) and PKM2 (**b**) was detected by RT‒qPCR. Data are the mean ± SD from three independent experiments with differences calculated using the delta-delta Ct method relative to the expression of the reference gene GAPDH. After treatment, cells were lysed, and extracted proteins were analyzed using Western blotting with β-actin as an internal control for MDA-MB-231 cells. Then, a representative Western blot shows the change in protein levels of LDHA (**c**) and PKM2 (**e**) compared to the loading control. Full-length blots are presented in Supplementary Figs. 5 and 6. The bar graph shows quantified protein levels (**d-f**). Thus, PKM2 activity was examined using an NADH/lactate dehydrogenase (LDH) coupled assay (**g**), in which the variation in OD at 340 nm indicates a variation in cell PKM2 activity. Each value represents the mean of three assays. Data are expressed as the mean ± SD of triplicates. ns; *p* > 0.05, *p < 0.05, **p < 0.01, ***p < 0.001, ****p < 0.0001 as indicated

Considering that upregulated PKM2 and LDHA, which are two crucial glycolytic enzymes, facilitate the growth advantage of tumor cells, and to determine the effect of ATRA on glycolysis in ER-positive and triple-negative cells, we examined the variation in the mRNA and protein expression of LDHA and PKM2, as well as PKM2 activity, in treated MCF-7 and MDA-MB-231 cells. In MCF-7 cells, we found that LDHA mRNA expression (Fig. 5. a) decreased significantly after treatment, mostly after the combination treatment (56.37%) (*p* < 0.0001); moreover, LDHA protein expression (Fig. 5. c-d) decreased significantly upon ATRA treatment without or with fulvestrant, by 63.53% (*p* < 0.01) and 40.63% (*p* < 0.05), respectively. Then, PKM2 variation after treatments was evaluated, and mRNA, protein, and activity expression decreased significantly. Hence, after inhibitor combination, mRNA expression (Fig. 5. b) decreased by 48.5% (*p* = 0.0002), protein expression (Fig. 5. e-f) decreased by 23% (*p* < 0.05), and PKM2 activity (Fig. 5. g) decreased by 32.81% (*p* = 0.003). However, MDA-MB-231 treated cells showed a significant increase in LDHA mRNA expression (Fig. 6. a), mostly after inhibitor combination (57.67%) (*p* = 0.0018), while there was no significant variation in LDHA protein expression (Fig. 6. c-d) after treatment. In addition, PKM2 mRNA expression (Fig. 6. b) increased significantly with ATRA or fulvestrant treatment, with no significant increase after the combination treatment. Furthermore, following the same conditions, PKM2 protein expression (Fig. 6. e-f) and activity (Fig. 6. g) indicated no significant modulation after treatments.

### ATRA combined with downregulated H19 or hTERT regulates H19 and telomerase expression in MCF-7 cells

**Fig. 7:**
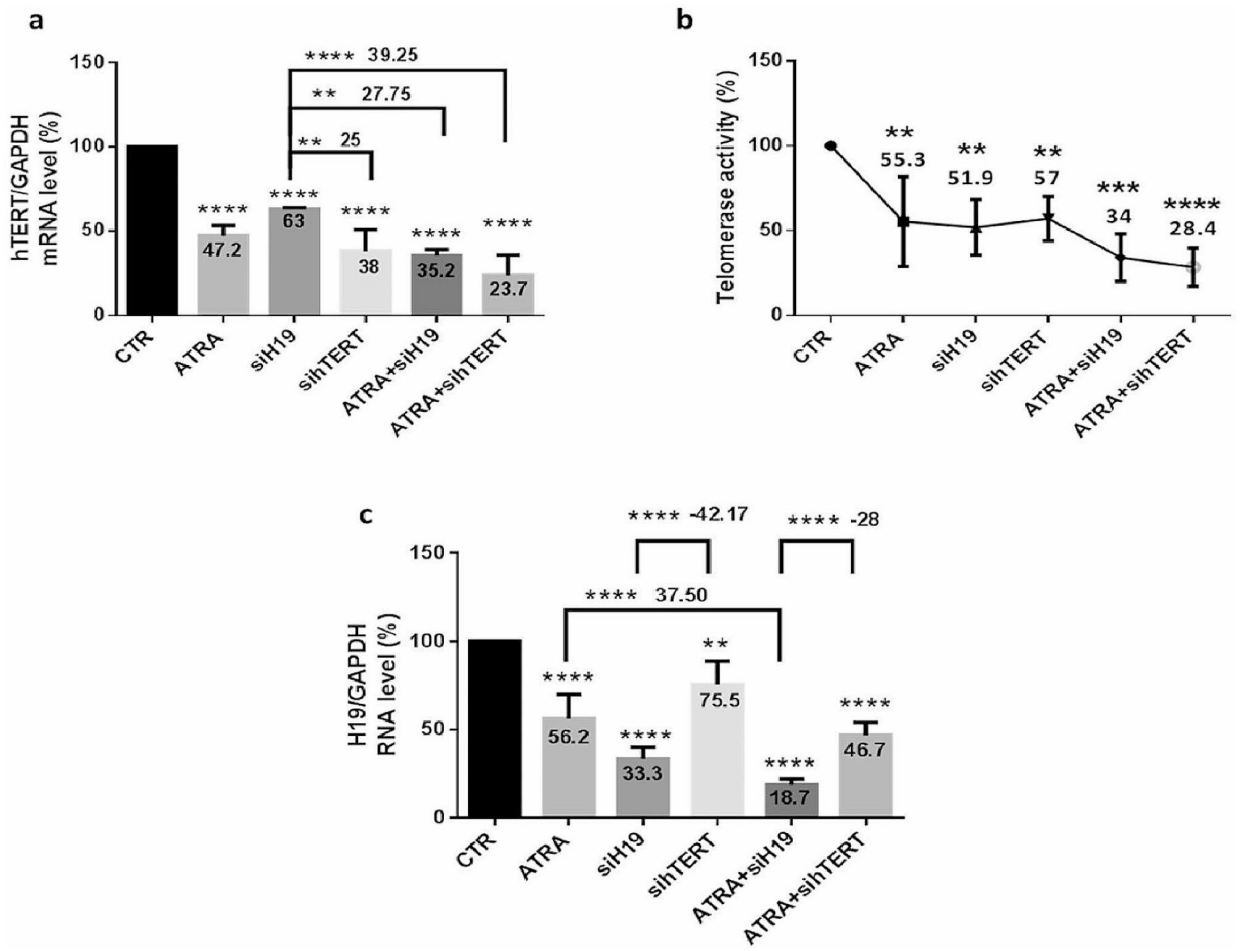
ATRA combined with downregulated H19 or hTERT regulates telomerase and H19 expression in MCF-7 cells. MCF-7 cells were seeded at a density of 2 × 10^6^ cells per dish. At 80% confluence, cells were treated with 5 µM of ATRA and/or transfected with H19 siRNA or hTERT siRNA using Hi-perfect transfection reagent. Then, cells were harvested for RNA extraction, followed by quantification performed by RT‒qPCR of the expression of hTERT (**a**) and H19 (**c**). Data are the mean ± SD from three independent experiments with differences calculated using the delta-delta Ct method relative to the expression of the reference gene GAPDH. After treatment, telomerase activity was compared to that of the control and detected using a qTRAP assay (**b**). Each value represents the mean of three assays. Data are expressed as the mean ± SD of triplicates. ns; *p* > 0.05, *p < 0.05, **p < 0.01, ***p < 0.001, ****p < 0.0001 as indicated

For a better understanding of ATRA importance and mechanism of action on BC cell development and to investigate the involvement of H19 and hTERT in these processes in MCF-7 cells, mainly after showing reduced H19 and hTERT expression caused by ATRA treatment, MCF-7 cells were treated with ATRA alone or coupled with H19 siRNA transfection first or with hTERT siRNA transfection second. Our data indicate a highly significant decrease in hTERT mRNA expression (Fig. 7. a), activity (Fig. 7. b), and H19 expression (Fig. 7. c) after cell transfection and treatment under all conditions, mainly after ATRA combination with siH19 or sihTERT. ATRA treatment combined with downregulated H19 significantly decreased hTERT mRNA expression by 64.8% (*p* < 0.0001), telomerase activity by 66% (*p* < 0.001), and H19 by 81.3% (*p* < 0.0001). Moreover, ATRA treatment combined with downregulated hTERT significantly decreased hTERT mRNA expression by 76.3% (*p* < 0.0001), telomerase activity by 71.6% (*p* < 0.0001), and H19 by 53.3% (*p* < 0.0001). In summary, the RNA expression pattern and telomerase activity obtained by gene silencing were similar to those obtained after treatment with the inhibitors.

### The implication of H19 and hTERT, in addition to ATRA, in the regulation of glycolytic enzymes in MCF-7 cells

**Fig. 8:**
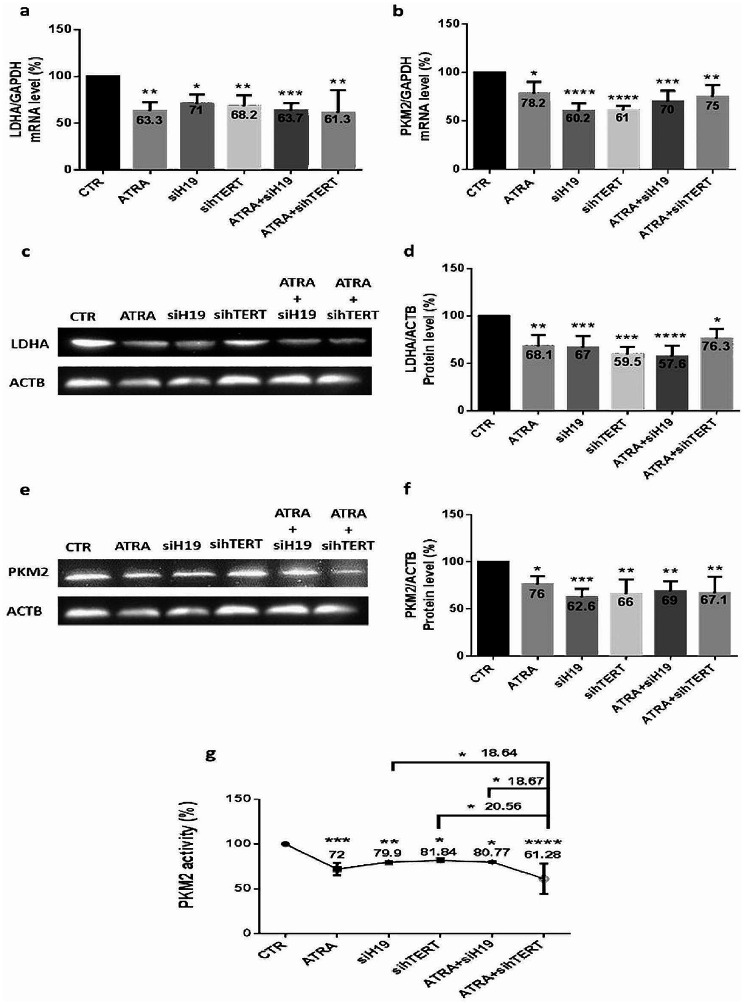
ATRA reduces glycolytic enzymes expression through H19 and hTERT in MCF-7 cells. After showing reduced H19 and hTERT expression caused by ATRA treatment in MCF-7 cells, 2 × 10^6^ cells were seeded and treated with 5 µM ATRA alone or coupled with siRNA transfection of H19 (20 nM) first or with hTERT siRNA (20 nM) second. Hence, cells were harvested for RNA extraction, followed by RT‒qPCR quantification of LDHA (**a**) and PKM2 (**b**) expression. Data are the mean ± SD from three independent experiments with differences calculated using the delta-delta Ct method relative to the expression of the reference gene GAPDH. Next, extracted proteins from transfected and/or treated cells were analyzed using Western blotting with β-actin as an internal control for MCF-7 cells. Representative Western blot showing the change in protein levels of LDHA (**c**) and PKM2 (**e**) compared to the loading control. Full-length blots are presented in Supplementary Figs. 7 and 8. The quantitative analysis of the intensity of the bands is shown in the bar graph (**d-f**). Furthermore, PKM2 activity was examined using an NADH/lactate dehydrogenase (LDH) coupled assay (**g**). Each value represents the mean of three assays. Data are expressed as the mean ± SD of triplicates. *p < 0.05, **p < 0.01, ***p < 0.001, ****p < 0.0001 as indicated

As mentioned above, ATRA downregulates glycolytic enzymes levels in MCF-7 cells. In the interest to determine whether H19 and hTERT are implicated in LDHA and PKM2 direct regulation, MCF-7 cells were treated with ATRA alone or coupled with H19 siRNA transfection first or with hTERT siRNA transfection second to determine whether the targeted inhibition of these genes could modulate glycolysis. Our results show a significant decrease in the mRNA and protein expression of LDHA and PKM2, as well as in PKM2 activity. In fact, as provoked by ATRA, LDHA mRNA expression (Fig. 8. a) decreased significantly after siH19 (29%) (*p* < 0.05) or sihTERT (31.8%) (*p* < 0.01) transfection; likewise, for ATRA combined with downregulated H19 or hTERT. A similar expression pattern was observed at the protein LDHA levels (Fig. 8. c-d). Similar to LDHA, PKM2 mRNA (Fig. 8. b) and protein (Fig. 8. e-f) expression, as well as PKM2 activity (Fig. 8. g), showed a highly significant decrease, nearly 35%, after treatment. Based on these results, a correlation among H19, hTERT, and glycolytic enzymes could be assessed.

### Overexpression of ER(α) or ER(β) modulates H19 and telomerase in MDA-MB-231 cells

**Fig. 9:**
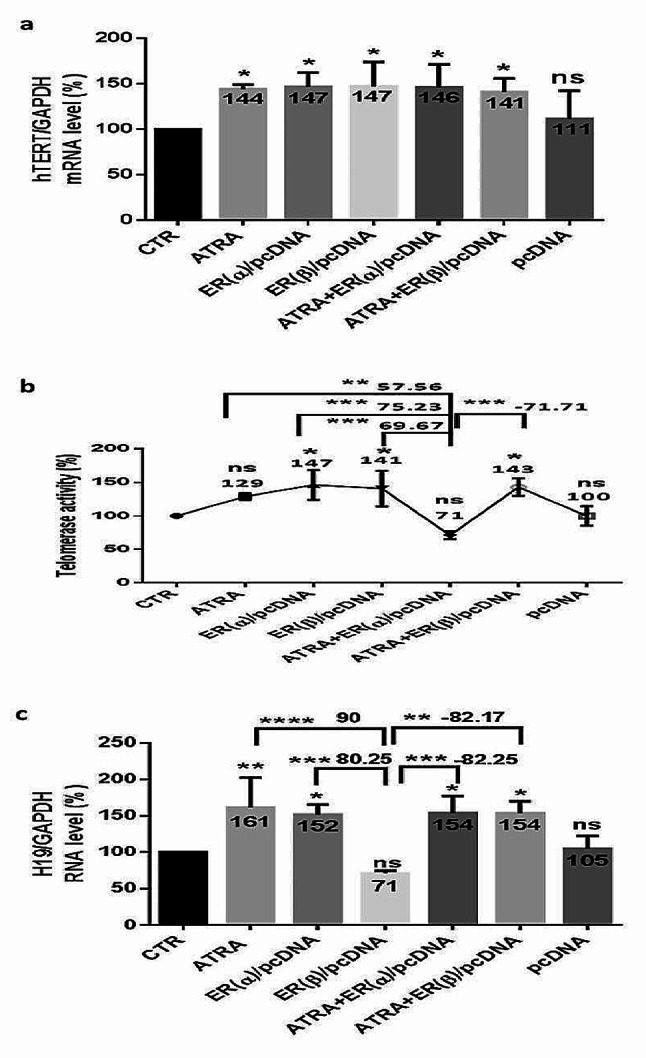
Estrogen receptor alpha or beta overexpression modulates H19 and telomerase in MDA-MB-231 cells. To clarify ER(α) and ER(β) function in regulating H19 and hTERT, MDA-MB-231 cells were transfected with ER(α) or ER(β) expression plasmids. MDA-MB-231 cells were seeded at a density of 1.5 × 10^6^ cells per dish, treated with 5 µM ATRA, and/or transfected with plasmid coding estrogen receptor alpha (ER(α)/pcDNA) or beta (ER(β)/pcDNA) expression for 48 h using the Attractene Transfection Reagent protocol. Then, RNA was extracted from cells and quantified for the expression of hTERT (**a**) and H19 (**c**). Data are the mean ± SD from three independent experiments with differences calculated using the delta-delta Ct method relative to the expression of the reference gene GAPDH. Following transfection and treatments, telomerase activity was detected using a qTRAP assay (**b**). Each value represents the mean of three assays. Data are expressed as the mean ± SD of triplicates. ns; *p* > 0.05, *p < 0.05, **p < 0.01, ***p < 0.001, ****p < 0.0001 as indicated

Considering that ER isoforms alpha and beta are involved in BC progression and glycolysis, to examine whether this biological process could occur through H19 and hTERT and with the aim of exploring whether ATRA could regulate this signaling pathway, we transfected treated or untreated MDA-MB-231 cells with ER(α) or ER(β) expression plasmids for 48 h. The outcome of ATRA treatment, ER(α) plasmid DNA transfection (ER(α)/pcDNA), ER(β) plasmid DNA transfection (ER(β)/pcDNA), and the combination of ATRA and each plasmid DNA transfection induced a significant increase in hTERT mRNA expression (Fig. 9. a), almost 45% (*p* < 0.05). Hence, telomerase activity (Fig. 9. b) increased significantly after ER(α) plasmid transfection (47%) (*p* < 0.05) or ER(β) plasmid transfection (41%) (*p* < 0.05) and after ATRA combination with ER(β) plasmid transfection (43%) (*p* < 0.05). However, telomerase activity showed no significant variation following ATRA treatment and after ATRA combined with ER(α) plasmid transfection. In addition, H19 (Fig. 9. c) was upregulated significantly by almost 55% following the aforementioned treatments, except for ER(β) plasmid transfection, in which H19 expression variation was nonsignificant.

### Effect of upregulated ER(α) or ER(β) in modulating LDHA and PKM2 enzymes in MDA-MB-231 cells

**Fig. 10:**
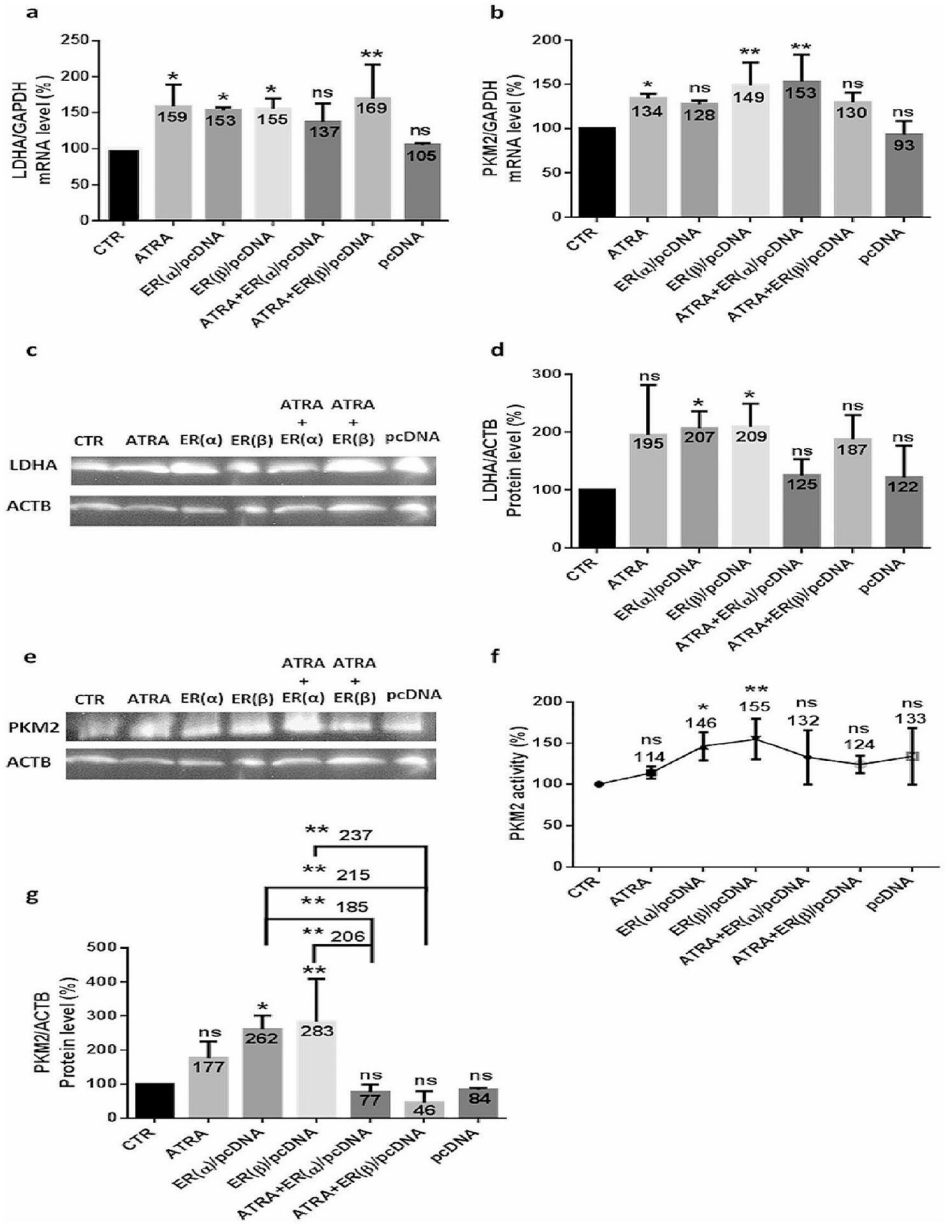
Upregulated estrogen receptor alpha or beta modulates LDHA and PKM2 in MDA-MB-231 cells. To find a direct relationship between estrogen receptors and glycolytic enzymes regulation, we transfected MDA-MB-231 cells with ER(α)/pcDNA or ER(β)/pcDNA plasmid and then evaluated LDHA and PKM2 expression variation. MDA-MB-231 cells were seeded, treated with 5 µM ATRA, and/or transfected for 48 h using the Attractene Transfection Reagent protocol. Thus, mRNA was quantified, and the expression of LDHA (**a**) and PKM2 (**b**) was detected by qPCR. Data are the mean ± SD from three independent experiments with differences calculated using the delta-delta Ct method relative to the expression of the reference gene GAPDH. Extracted proteins were analyzed using Western blotting with β-actin as an internal control for MDA-MB-231 cells. Representative Western blot showing the change in protein levels of LDHA (**c**) and PKM2 (**e**) compared to the loading control. Full-length blots are presented in Supplementary Figs. 9 and 10. The quantitative analysis of the intensity of the bands is shown in the bar graph (**d-g.**) Afterwards, PKM2 activity was examined using an NADH/lactate dehydrogenase (LDH) coupled assay (**f**). Each value represents the mean of three assays. Data are expressed as the mean ± SD of triplicates. ns; *p* > 0.05, *p < 0.05, **p < 0.01, ***p < 0.001, ****p < 0.0001 as indicated

By taking into account that ER(α) overexpression is related to increased proliferation and metastasis in BC, while ER(β) function remains elusive, it may have a bi-faceted role in BC, based on our results showing that H19 and telomerase levels increase after ER(α) or ER(β) overexpression and considering that the latter can be involved in glycolysis modulation, ATRA treated or untreated MDA-MB-231 cells, triple-negative cells, were transfected with ER(α) or ER(β) expression plasmids for 48 h. Subsequently, LDHA and PKM2 regulation after transfection and treatment was detected. Similar to the effect caused by ATRA, LDHA mRNA expression (Fig. 10. a) increased significantly following ER(α) plasmid transfection (53%) (*p* < 0.05), ER(β) plasmid transfection (55%) (*p* < 0.05), and after ATRA combined with ER(β) plasmid transfection (69%) (*p* < 0.01); however, no significant variation was observed after ATRA combination with ER(α) plasmid transfection. Thus, LDHA protein (Fig. 10. c-d) expression increased significantly only after ER(α) or ER(β) transfection by almost 108% (*p* < 0.05). Interestingly, as presented for ATRA, PKM2 mRNA (Fig. 10. b) presented a highly significant increase after ER(α) or ER(β) transfection by 49% and 53%, respectively (*p* < 0.01). Only after ER(α) or ER(β) transfection, PKM2 protein (Fig. 10. e-g) expression and activity (Fig. 10. f), increased significantly, while nonsignificant variation was indicated after the other treatments and combinations.

### ATRA regulates miR-let-7a through H19 and hTERT in MCF-7 and MDA-MB-231 cells

**Fig. 11:**
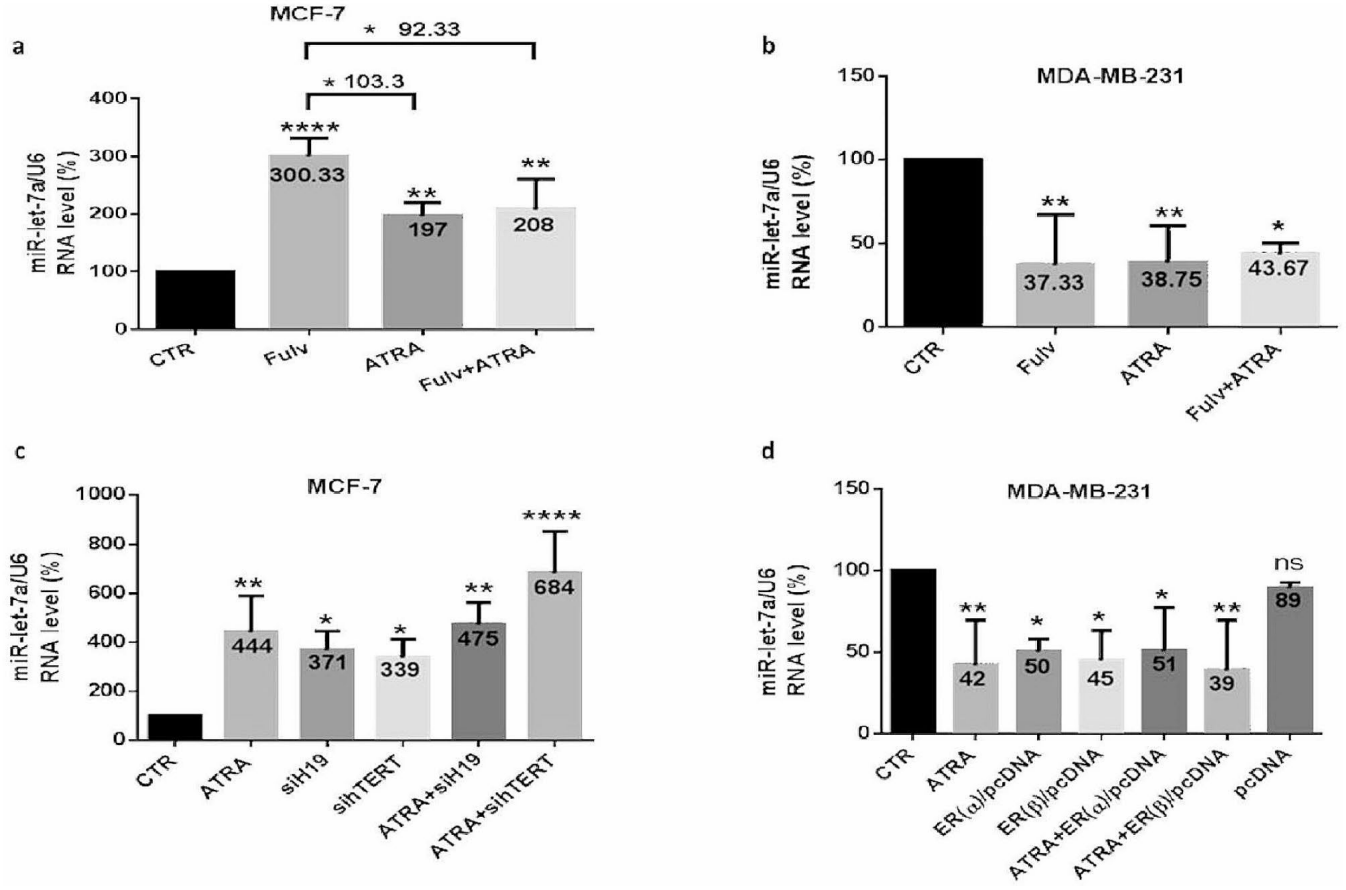
ATRA regulates miR-let-7a via H19 and hTERT in MCF-7 and MDA-MB-231 cells. To examine miR-let-7a implication in glycolytic enzymes regulation through H19 and hTERT, MCF-7 and MDA-MB-231 cells were seeded and subjected to the same conditions of treatments and transfections aforementioned for the two cell lines. Thus, miR-let-7a was quantified by Q-PCR, and its modulation was evaluated in MCF-7 cells treated with ATRA and/or fulvestrant (**a**) and in MDA-MB-231 cells treated with ATRA and/or fulvestrant (**b**). Next, miR-let-7a regulation was evaluated after ATRA-treated or untreated MCF-7 cells were transfected with siH19 or sihTERT (**c**) and after ATRA-treated or untreated MDA-MB-231 cells were transfected with ER(α) or ER(β) plasmid expression (**d**). Each value represents the mean of three assays. Data are expressed as the mean ± SD of triplicates. ns; *p* > 0.05, *p < 0.05, **p < 0.01, ***p < 0.001, ****p < 0.0001 as indicated

Given that miR-let-7a acts as a tumor suppressor by targeting some genes to affect signaling pathways by binding to the mRNA sequences, resulting in translational repression and mRNA degradation, we investigated miR-let-7a modulation in MCF-7 and MDA-MB-231 following ATRA treatment and transfections aforementioned for the two cell lines. MiR-let-7a RNA expression was quantified using the primer sequences mentioned in Table 2. Interestingly, in MCF-7 cells, miR-let-7a indicated a highly significant increase after ATRA and/or fulvestrant (Fig. 11. a); however, miR-let-7a decreased strongly and significantly following the same treatments in MDA-MB-231 cells (Fig. 11. b). Moreover, ATRA-treated or untreated MCF-7 cells transfected with siH19 or sihTERT showed a highly significant increase in miR-let-7a, mostly after the ATRA and siH19 combination (375%) (*p* < 0.001), as well as after the ATRA and sihTERT combination (584%) (*p* < 0.0001) (Fig. 11. c). However, ATRA-treated or untreated MDA-MB-231 cells transfected with ER(α) or ER(β) plasmid showed a highly significant decrease in miR-let-7a, almost 50%, under all conditions executed (Fig. 11. d).

## Discussion

Having a better characterization of the known and newly discovered potential markers would be of importance for the care and treatment of breast cancer [43]. LncRNAs particularly H19 [44, 45] and hTERT [16] are important biomarkers in breast cancer based on their main roles in glycolysis [46, 47]. Indeed, retinoic acid has inhibitory effects on proliferation and cancer cell migration by targeting cell proliferation proteins, such as epidermal growth factor receptor (EGFR) and vascular endothelial growth factor (VEGF) [41]. Consequently, the ultimate aim of our study is to investigate a possible relationship between H19, hTERT, and glycolytic metabolism that could be modulated by ATRA in breast cancer. In particular, we focused on the modulation of the expression and activity of PKM2 and the expression of LDHA in the glycolysis pathway, as well as, on the expression of miR-let-7a in MCF-7 and MDA-MB-231 breast cancer cell lines. As reported by Prat et al. [48], the effect of ATRA on breast cancer may be linked to the heterogeneity of this tumor; thus, the identification of specific markers defining breast cancer subtypes with particular sensitivity to ATRA represents a priority in our study. We first assessed the effect of ATRA on ER(α) and ER(β) mRNA expression in MCF-7 cells; however, H19 and hTERT were evaluated in both cell lines. Our experiments demonstrated that ER(α), H19, and hTERT RNA expression was reduced in MCF-7 cells compared to control cells, whereas ER(β) expression was increased, while in MDA-MB-231 cells, H19 and hTERT expression was increased. These differences confirm the fact that retinoids are known to affect hormone-dependent breast cancer cells only [49]. Recent studies have revealed that ATRA in combination with anti-tumor agents holds promise to enhance and improve anti-carcinogenic therapies [50]. In fact, combining ATRA with ER inhibitors such as tamoxifen inhibits growth and induces apoptosis of breast cancer cells [51]. Using the previous conditions, the combinatory effect of ATRA and fulvestrant on MCF-7 and MDA-MB-231 cells was evaluated. Regarding the expression of genes, the results were discordant between the two cell types. We found that in MCF-7 cells, following ATRA and/or fulvestrant treatment, the expression of ER(α), H19, hTERT, PKM2, and LDHA was reduced, as well as PKM2 activity and telomerase activity. However, ER(β) and miR-let-7a expression was increased. ER(α) is well known to be upregulated in the majority of breast cancers; it stimulates cancer cell proliferation, and its expression is a hallmark of hormone-dependent tumor growth [52]. Over the years, much evidence has shown the vital effect of ER(β) in breast cancer. Although there is controversy among scientists. ER(β) is generally thought to have antiproliferative effects in disease progression. In fact, the structure of ER(β) is homologous to that of ER(α), suggesting that while ER(β) could bind the same target genes as ER(α), it might have different specific ligands [53]. The role of ER(β) in BC initiation and proliferation has not yet been clearly established. In fact, several studies have suggested and demonstrated that ER(β) inhibits the proliferation, migration, and invasion of BC cells [54]; thus, ER(β) exhibits an inhibitory action on ER(α) mediated gene expression and, in many instances, opposes the actions of ER(α) [55]. Moreover, the expression of ER(β) may be regulated by DNA methylation, a reaction that is catalyzed by DNA methyltransferase (DNMT). Inhibition of DNA methyltransferase (DNMT) by fulvestrant increased the levels of ER(β), which exerted similar potency on DNMT activity as made by DNMT inhibitor [56]. This finding is in line with our study, where ATRA or/and fulvestrant reduced ER(α) and increased ER(β) expression. Thereafter, in this regard, it can be expected that these disorders caused by ATRA are accompanied by a change in upregulated oncogenes, tumor biomarkers, and glycolysis enzymes. As proven by SUN et al., H19 knockdown in MCF-7 cells resulted in a decrease in viable cell number and a blockade of estrogen-induced cell proliferation, indicating that H19 plays a significant role in cell survival and estrogen-induced cell proliferation in MCF-7 cells [10]. Second, increased telomerase activity and hTERT expression are reported in almost all human malignancies [57]. Recent studies have shown that certain miRNA expression correlate with tumor aggressiveness, and treatment responses suggesting that miRNAs can be used as diagnostic or prognostic markers [58]. Thereafter, dysregulated miRNA expression is frequently associated with the development of many types of human tumors, of which reduced expression of let-7 miRNA has been reported in breast cancer [59]. As discussed by Howard et al., let-7 miRNA is considered to be regulated by estrogen via ER(α), and estrogen signaling has been shown to regulate let-7 miRNA through direct ER(α) binding site interactions in estrogen receptor-positive breast cancer cells [60]. In this context, an increase in miR-let-7a expression upon ATRA treatment in MCF-7 cells could suppress the expression of several cancer-related genes in breast cancer, subsequently affecting biological processes such as glycolysis [61]. PKM2 is upregulated in breast cancer and can regulate tumor progression by promoting tumor cell viability, indicating thereafter that PKM2 is a potentially therapeutic target in breast cancer. YAO et al. have shown that miRNA let-7a can induce breast cancer cell apoptosis and inhibit cell proliferation, migration, and invasion; therefore, miR-let-7a inhibits aerobic glycolysis and proliferation of breast cancer cells by inhibiting PKM2 expression [36]. Together with our results, CHU et al. reported that the knockdown of PKM2 decreases the activity of pyruvate kinase in adenocarcinoma cells, and Shikonin which represents a novel PKM2 inhibitor, reduced PKM2 activity, which decreases cancer cell proliferation and survival [62]. Furthermore, Shikonin inhibits the rates of cellular lactate production and glucose consumption, in which LDHA plays a crucial role. Similar to PKM2, the regulation of LDHA is critical in cancer cells. One study showed that targeting LDHA with siRNA or small molecule inhibitors increased oxygen consumption and reactive oxygen species production, reduced glucose uptake and lactate production, and decreased tumor cell growth [32]. Afterward, miR-let-7a could be considered a microRNA that acts as a potent regulator due to its known role in regulating glycolysis in cancer cells. Taken together, our experiments demonstrated that ATRA regulates PKM2 and LDHA via miR-let-7a by inhibiting H19 and hTERT expression through estrogen receptors in MCF-7 cells. Besides, for MDA-MB-231 cells, upon ATRA and/or fulvestrant treatment, H19, hTERT, LDHA, and PKM2 RNA expression increased, while miR-let-7a expression decreased. The same treatment did not show a significant variation either on LDHA and PKM2 protein expression, or on PKM2 and telomerase activity. Increased expression of previously described RNAs after treatment is reported by the MDA-MB-231 cell line response caused by resistance to ATRA treatment. Liu et al. demonstrated that multidrug resistance is a major problem in successful cancer chemotherapy, leading to gene and enzymes overexpression [63]. As previously mentioned, the expression of miR-let-7a was significantly lower in breast cancer cells than in corresponding adjacent normal tissues, which suggested that miR-let-7a downregulation was associated with the development of breast cancer. Based on our results, ATRA and/or fulvestrant decreased miR-let-7a expression, thereby activating glycolysis, which induced an increase in PKM2 and LDHA mRNA expression. Nonsignificant protein expression of PKM2 and LDHA, neither on PKM2 activity was shown. However, the increase of these genes at the mRNA level could be due to post-transcriptional regulation that regulate cancer progression [64]. Interestingly, despite the ATRA effect on RNA level gene variation, no significant effect was observed on protein levels in MDA-MB-231 cells. To further investigate the direct implication of H19 and hTERT in miR-let-7a and glycolysis regulation in MCF-7 cells, mainly after their inhibition upon ATRA treatment, siRNA knockdown of each of the previous molecules alone or coupled with ATRA treatment was performed. After MCF-7 cells were treated with ATRA alone or coupled with H19 siRNA transfection or with hTERT siRNA transfection, a highly significant decrease in H19, hTERT, PKM2, and LDHA expression, as well as in PKM2 and telomerase activities were detected. However, miR-let-7a expression was increased. Interestingly, regulated expression patterns obtained by gene silencing were similar to those obtained after treatment with ATRA and/or fulvestrant, indicating that ATRA induces an inhibitory effect on PKM2 and LDHA, with an increase in miR-let-7a expression, via H19 and hTERT. Our results are compatible with Kallen et al. who confirmed that H19 antagonizes let-7 microRNAs, in which it modulates let-7 availability by acting as a molecular sponge, affecting, thereafter, the expression of endogenous let-7 targets [65]. In addition, Hrdlicˇkova´ et al. reported that hTERT is regulated by multiple miRNAs, such as let-7 g, that regulates hTERT expression and decreases telomerase activity [66]. These results are in line with our findings in which hTERT knockdown increased miR-let-7a expression. Moreover, telomerase mRNA and activity decrease after H19 inhibition and vice versa, indicating the presence of interconnection between these aforementioned tumor biomarkers. This interconnection was demonstrated by El Hajj et al., where telomerase was regulated by H19 in human acute promyelocytic leukemia cells [23]. As previously described, the implication of ER(α) and ER(β) in the modulation of the expression of tumor biomarkers and glycolysis has been demonstrated. To further evaluate their function in MDA-MB-231 cells, treated or untreated cells were transfected with ER(α) or ER(β) expression plasmids. Overexpression of ER(α) or ER(β) is directly related to an increase in PKM2 and LDHA expression, while ATRA combined with ER(α) or ER(β) overexpression restored PKM2 and LDHA expression. Indeed, JavanMoghadam et al. demonstrated that ER(α) modulates breast cancer cell proliferation by regulating events during the S and G2/M phases of the cell cycle [67]. These findings are consistent with our results showing that ER(α) promotes glycolysis enzymes expression in MDA-MB-231 cells, thus, enhancing other biological processes, implicated in cancer cell progression. Contrary to the results observed in MCF-7 cells concerning ER(β) function, in MDA-MB-231 cells, ER(β) promotes glycolysis. This goes in Iine with our previous interpretation that ER(β) may have a bi-faceted role in breast cancer. Mishra et al. reported that the alteration in the expression of ER(α)/ER(β) balance is a critical step in breast cancer development and progression; the role of ER(β) in breast cancers expressing ER(β) alone, without ER(α), is less clear to date [56].

## Conclusions

**Fig. 12:**
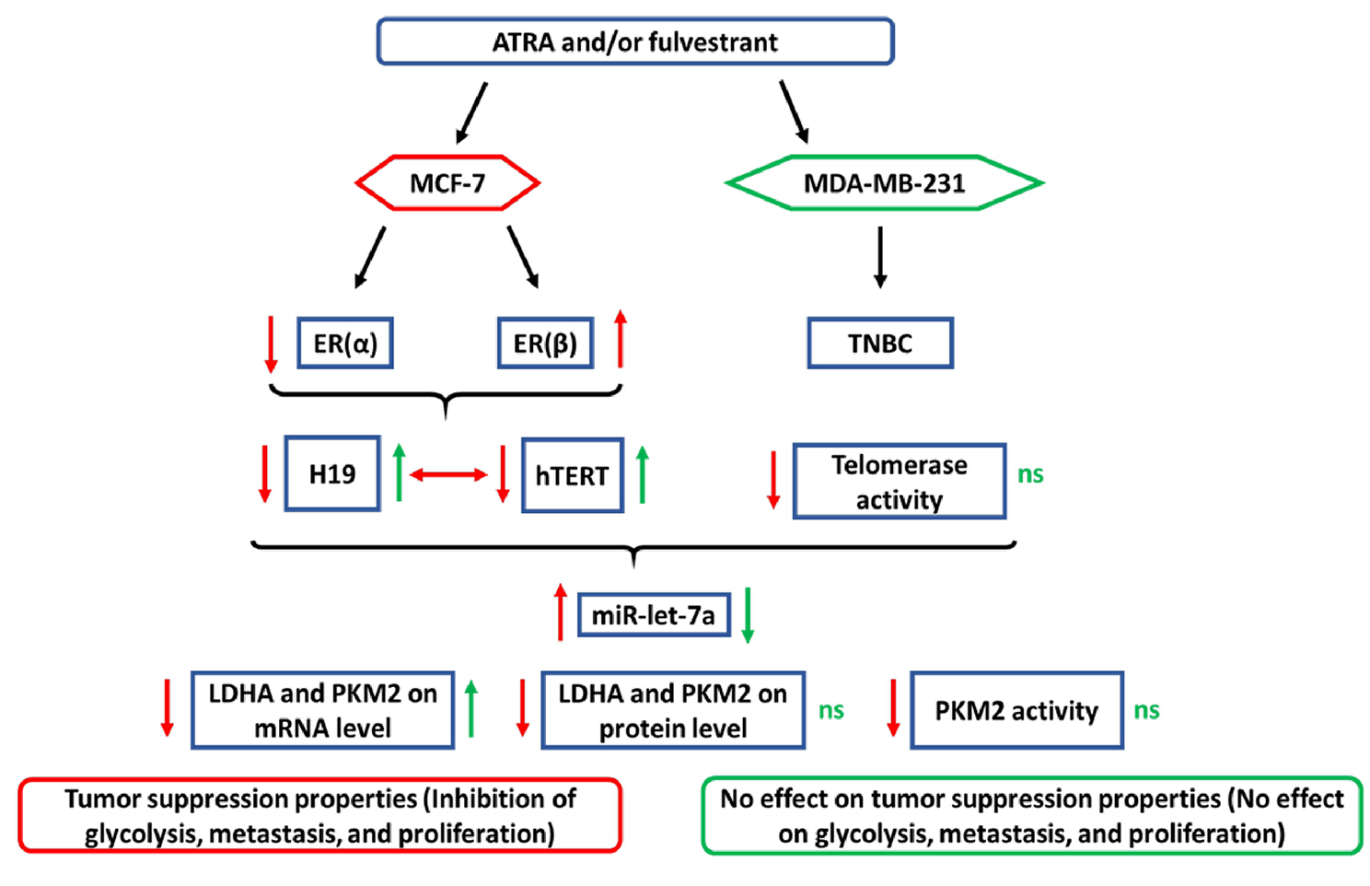
Summary diagram showing the variation in results after ATRA and/or fulvestrant treatment of MCF-7 and MDA-MB-231 cells. MCF-7 cell treatment with ATRA alone or coupled with fulvestrant inhibited PKM2 and LDHA, increased miR-let-7a, and inhibited H19 and hTERT expression by modulating ER(α) and ER(β). In addition, an interconnection between H19 and hTERT is noted, with a direct regulation carried out by each of them on miR-let-7a, PKM2, and LDHA. Effectively, increased expression of miR-let-7a and reduced expression of PKM2 and LDHA obtained by gene silencing were similar to those obtained after treatment with ATRA and/or fulvestrant. However, no significant variation in glycolytic enzymes expression or telomerase activity was detected in MDA-MB-231 cells upon the same treatment

The present study investigated the effect of ATRA on H19, telomerase, miR-let-7a, PKM2, and LDHA in MCF-7 and MDA-MB-231 cells, which are ER-positive and triple-negative cells, respectively. Our study elucidates a signaling pathway regulated by ATRA in breast cancer cells. Indeed, we confirmed that MCF-7 cell treatment with ATRA alone or coupled with fulvestrant inhibited PKM2 and LDHA, increased miR-let-7a, and inhibited H19 and hTERT expression by modulating estrogen receptors alpha and beta, with an interconnection between H19 and hTERT. However, no significant regulation of glycolytic enzymes or telomerase activity was detected in MDA-MB-231 cells upon the same treatment (Fig. 12). These results highlight that ATRA acts as a tumor suppressor, demonstrating therapeutic potential with its combination with fulvestrant in ER-positive cells. Further investigations are required to clarify the effect of ATRA on ER(β) isoforms, to assess direct binding sites of miR-let-7a on the other tumor biomarkers and to evaluate metastasis and invasion of cancer cells after treatment. Finally, these in vitro observations should be validated using an in vivo model with ATRA and fulvestrant combination for the treatment of breast cancer and presenting afterwards an advantage in BC patient’s response to fulvestrant.

### Electronic supplementary material

Below is the link to the electronic supplementary material.

Supplementary Material 1

## Data Availability

The datasets used and/or analyzed during the current study are available from the corresponding author upon reasonable request.

## Abbreviations

ACTB: Actin Beta
ATCC: American Type Culture Collection
ATP: Adenosine triphosphate
ATRA: *All-trans*-Retinoic acid
β-actin: Beta actin
BC: Breast cancer
BCA: Bicinchoninic acid
bp: Base pair
cDNA: Complementary DNA
CO_2_: Carbon dioxide
Ct: Cycle threshold
CTR: Control
DMEM: Dulbecco’s modified Eagle’s medium
DNA: Deoxyribonucleic acid
ECL: Enhanced chemiluminescence
ER: Estrogen Receptor
ER(α): Estrogen receptor alpha
ER(β): Estrogen receptor beta
ER(α)/pcDNA: Estrogen receptor alpha plasmid coding DNA
ER(β)/pcDNA: Estrogen receptor beta plasmid coding DNA
FBS: Fetal Bovine Serum
Fulv: Fulvestrant
GAPDH: Glyceraldehyde 3-phosphate dehydrogenase
HER2: Human epidermal receptor 2
hTERT: Human telomerase reverse transcriptase
IGF2: Insulin-like growth factor 2
kb: Kilobase
LB: Lysogeny broth
LDHA: Lactate dehydrogenase A
lncRNA: long non-coding RNA
miRNA: MicroRNA
MiR-let-7a: MicroRNA let-7a
mRNA: Messenger RNA
NADH: Nicotinamide adenine dinucleotide
ns: Nonsignificant
OD: Optical density
PEP: Phosphoenolpyruvate
PK: Pruvate kinase
PKM2: Pyruvate kinase M2
PMSF: Phenylmethylsulfonyl fluoride
PR: Progesterone receptor
PS: Penicillin/Streptomycin
RNA: Ribonucleic acid
RT‒PCR: Reverse-transcription polymerase chain reaction
siH19: Small interfering RNA of H19
sihTERT: Small interfering RNA of hTERT
siRNA: Small interfering RNA
TNBC: Triple-negative breast cancer
qPCR: Quantitative polymerase chain reaction
qTRAP: Quantitative telomeric repeat amplification protocol
