# Supplementary material for: All-trans-retinoic acid modulates glycolysis via H19 and telomerase: the role of mir-let-7a in estrogen receptor-positive breast cancer cells
Source: BMC Cancer. 2024 May 21;24:615. doi: 10.1186/s12885-024-12379-3 (PMC11106948; doi:10.1186/s12885-024-12379-3)
Supplement: Supplementary file 1 — Supplementary Material 1 [file 12885_2024_12379_MOESM1_ESM.pdf]

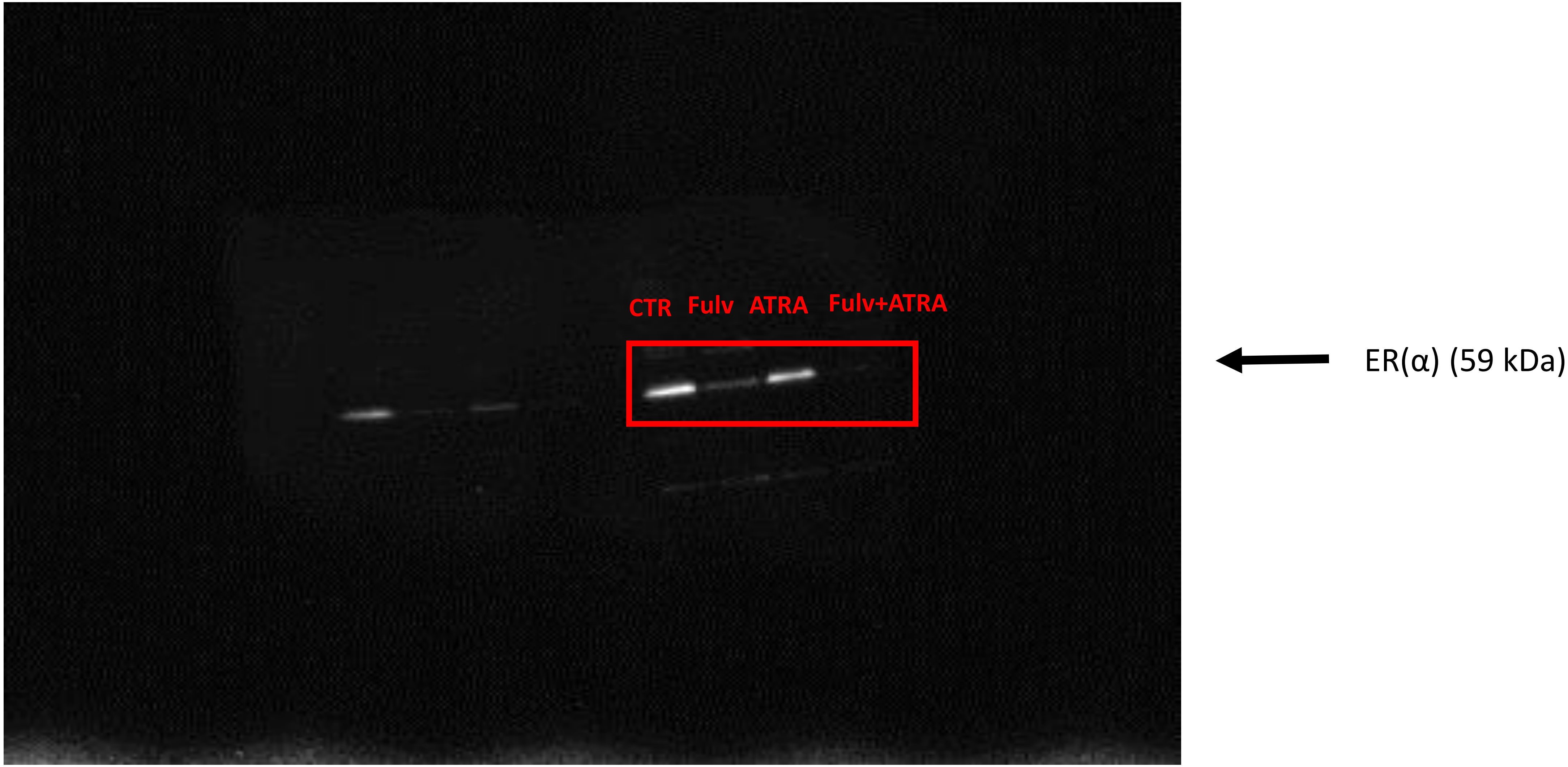

Supplementary fig 1. ATRA and/or fulvestrant modulates ER(α) in MCF-7 cells. (Full-length blots of fig 3.c)

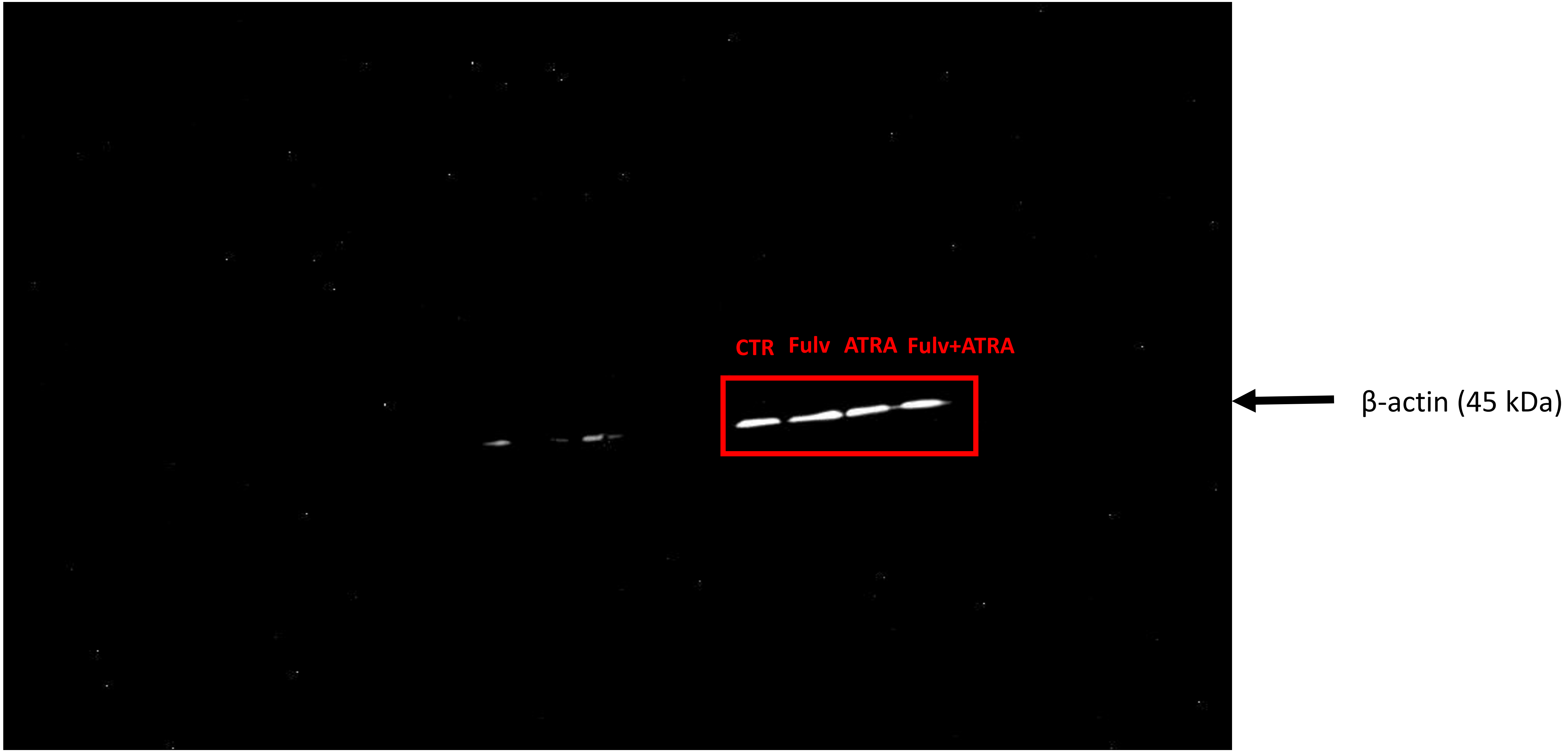

Supplementary fig 1. ATRA and/or fulvestrant modulates ER(α) in MCF-7 cells. (Full-length blots of fig 3.c)

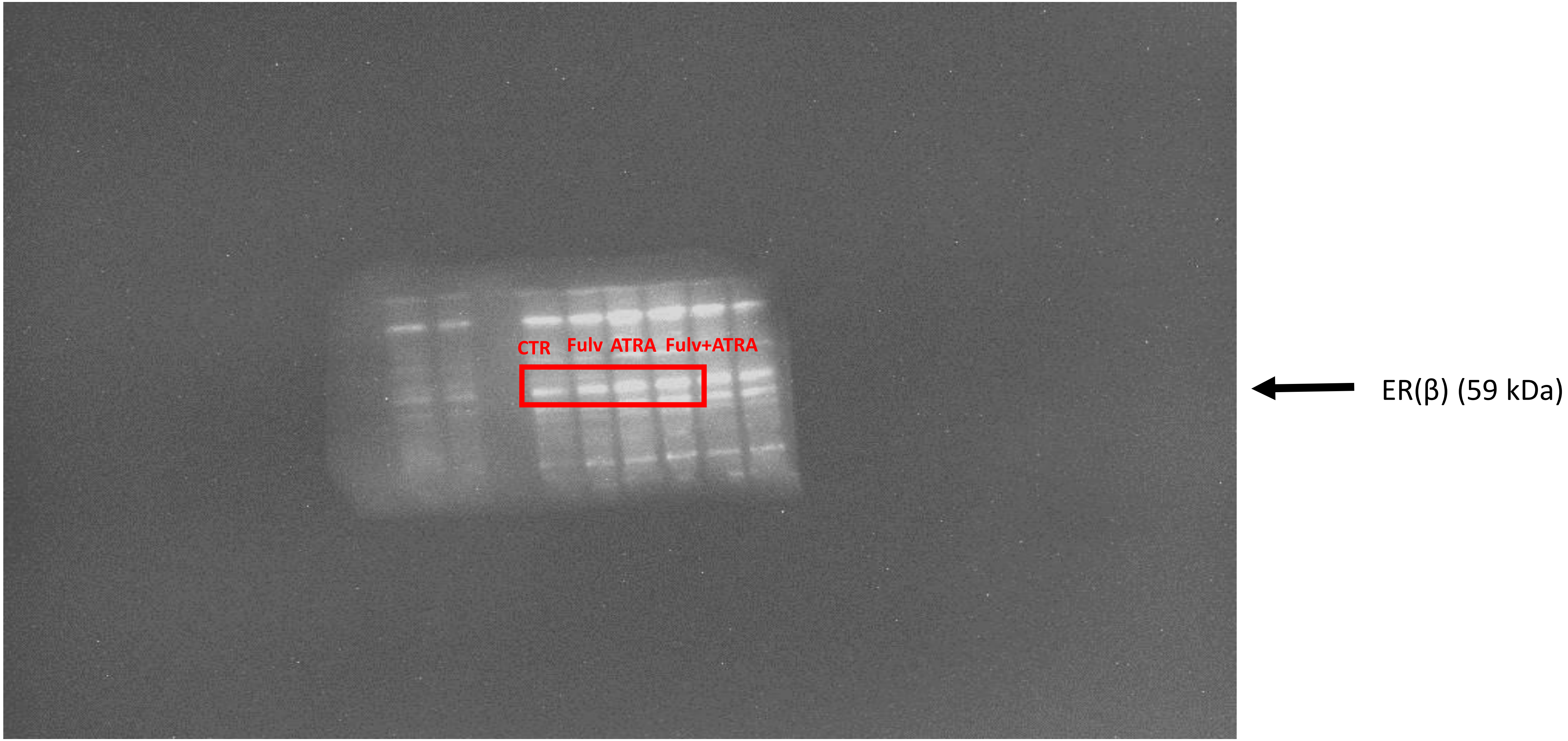

Supplementary fig 2. ATRA and/or fulvestrant modulates ER(β) in MCF-7 cells. (Full-length blots of fig 3.e)

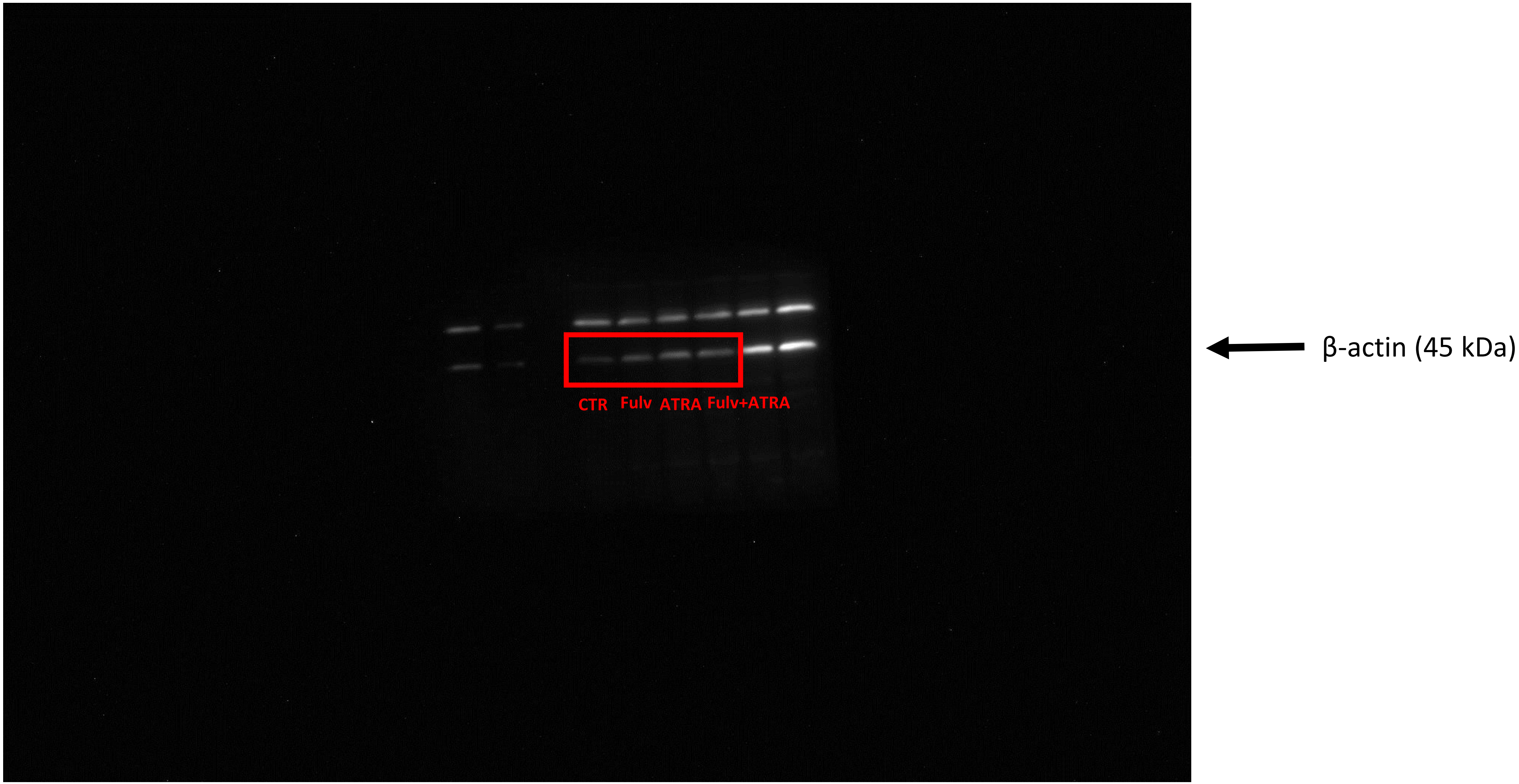

Supplementary fig 2. ATRA and/or fulvestrant modulates ER(β) in MCF-7 cells. (Full-length blots of fig 3.e)

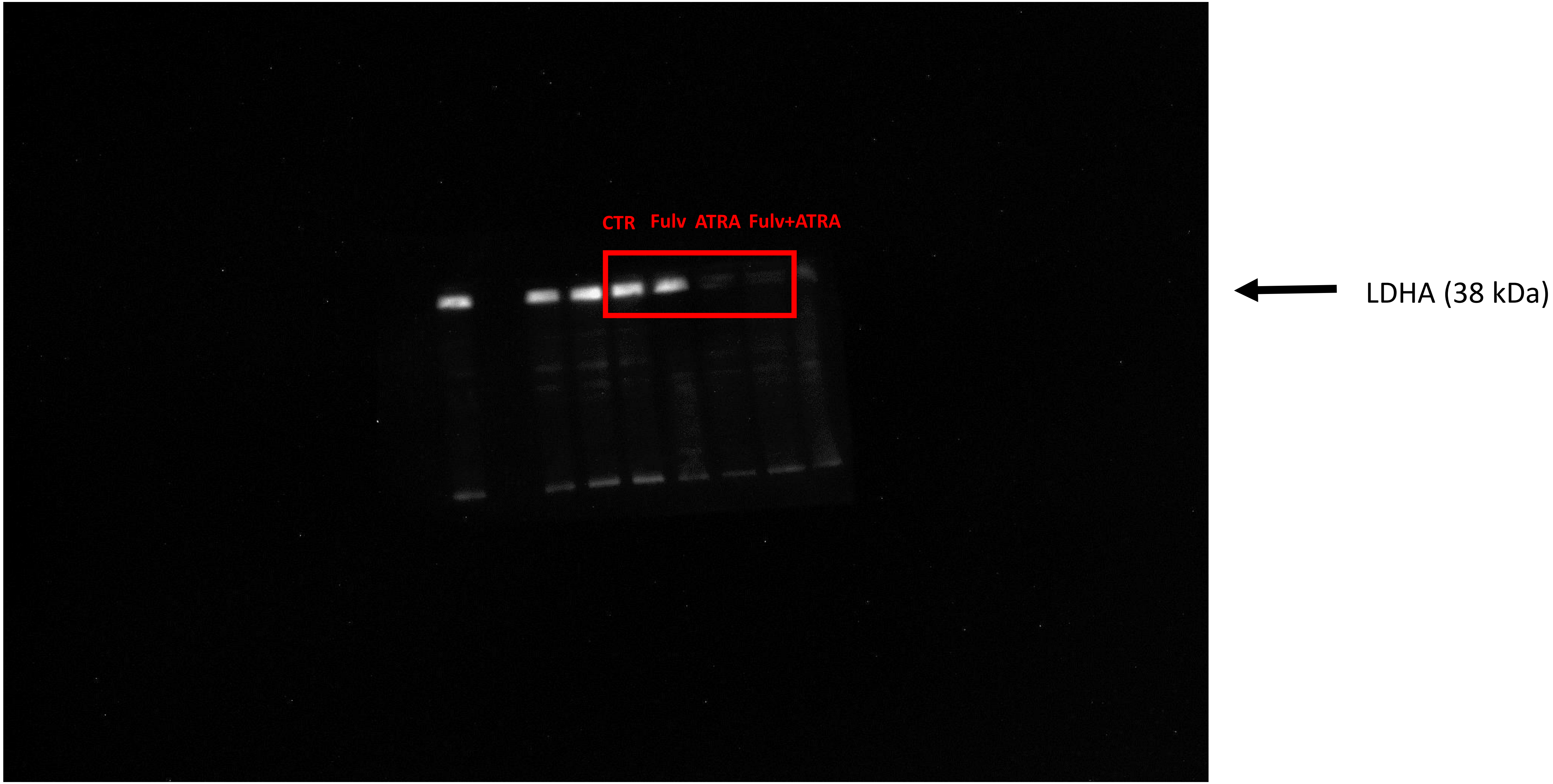

Supplementary fig 3. ATRA and/or fulvestrant modulates LDHA glycolytic enzyme in MCF-7 cells. (Full-length blots of fig 5.c)

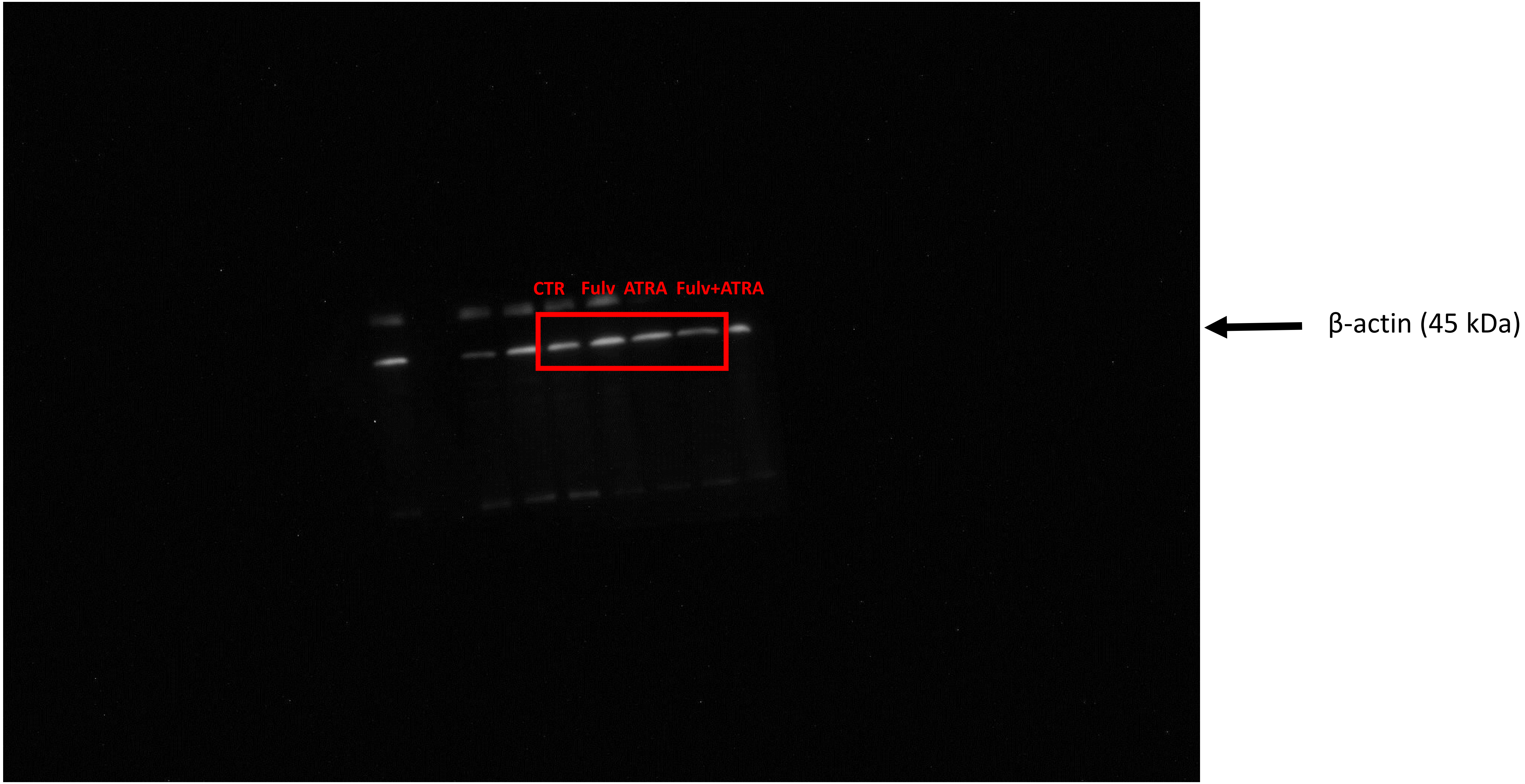

Supplementary fig 3. ATRA and/or fulvestrant modulates LDHA glycolytic enzyme in MCF-7 cells. (Full-length blots of fig 5.c)

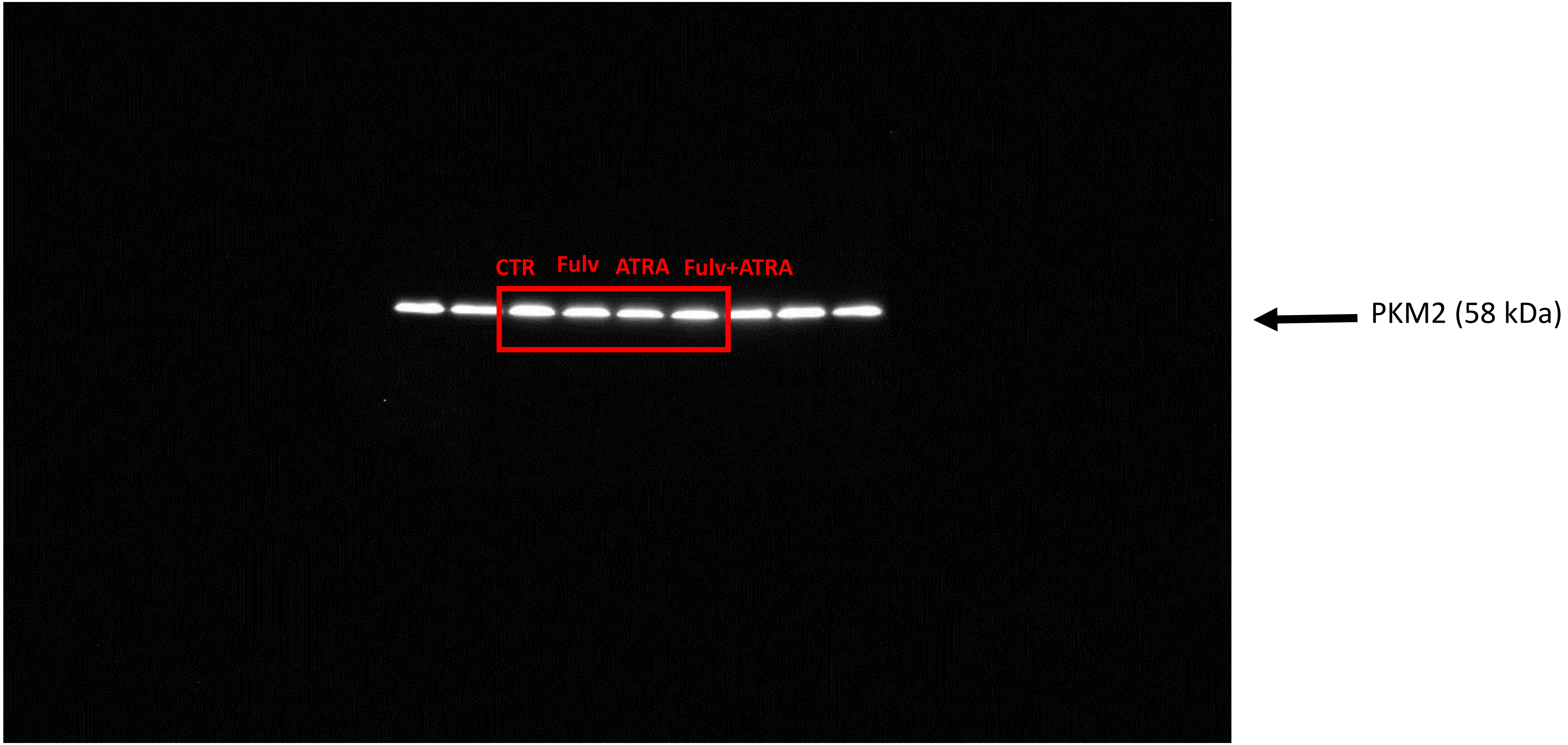

Supplementary fig 4. ATRA and/or fulvestrant modulates PKM2 glycolytic enzyme in MCF-7 cells. (Full-length blots of fig 5.e)

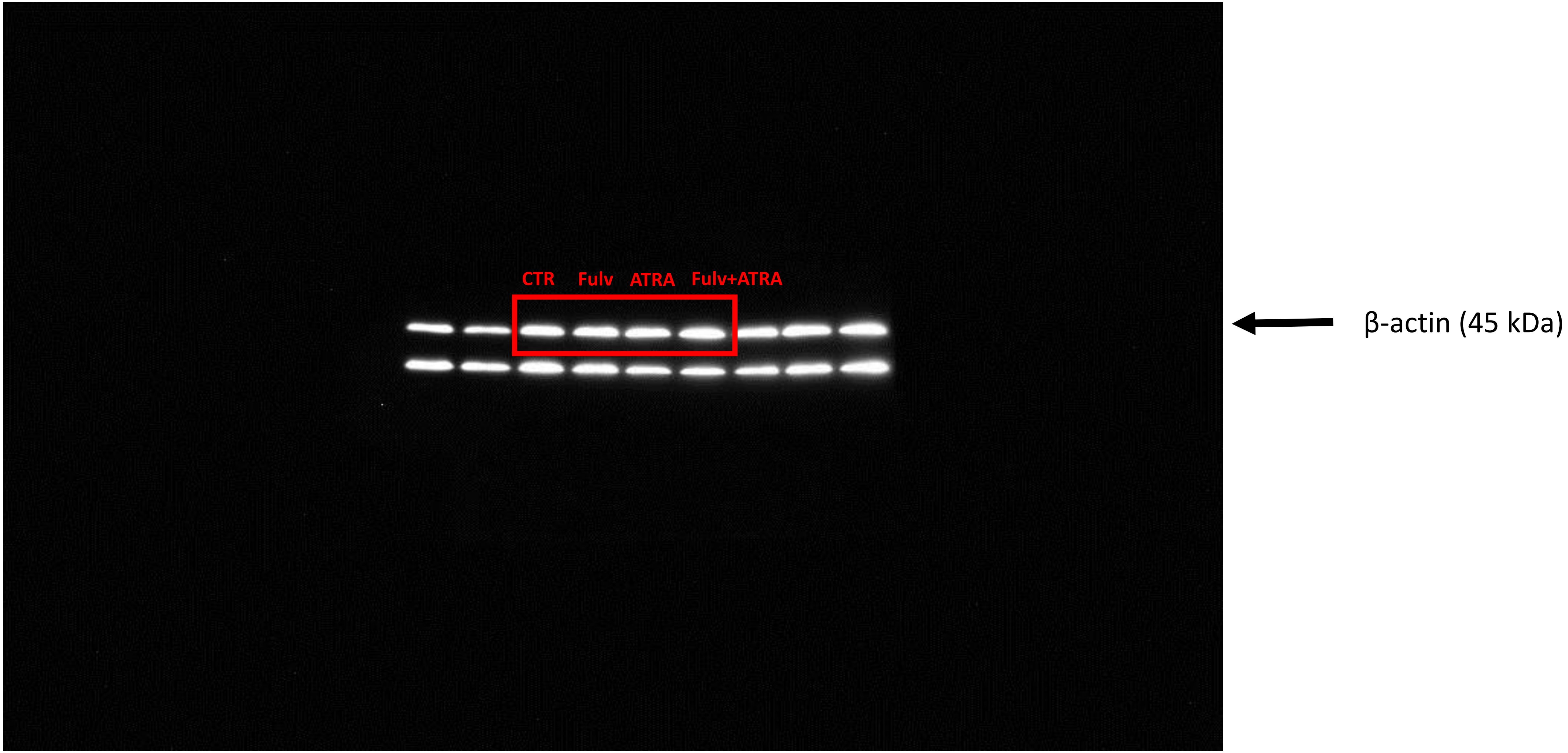

Supplementary fig 4. ATRA and/or fulvestrant modulates PKM2 glycolytic enzyme in MCF-7 cells. (Full-length blots of fig 5.e)

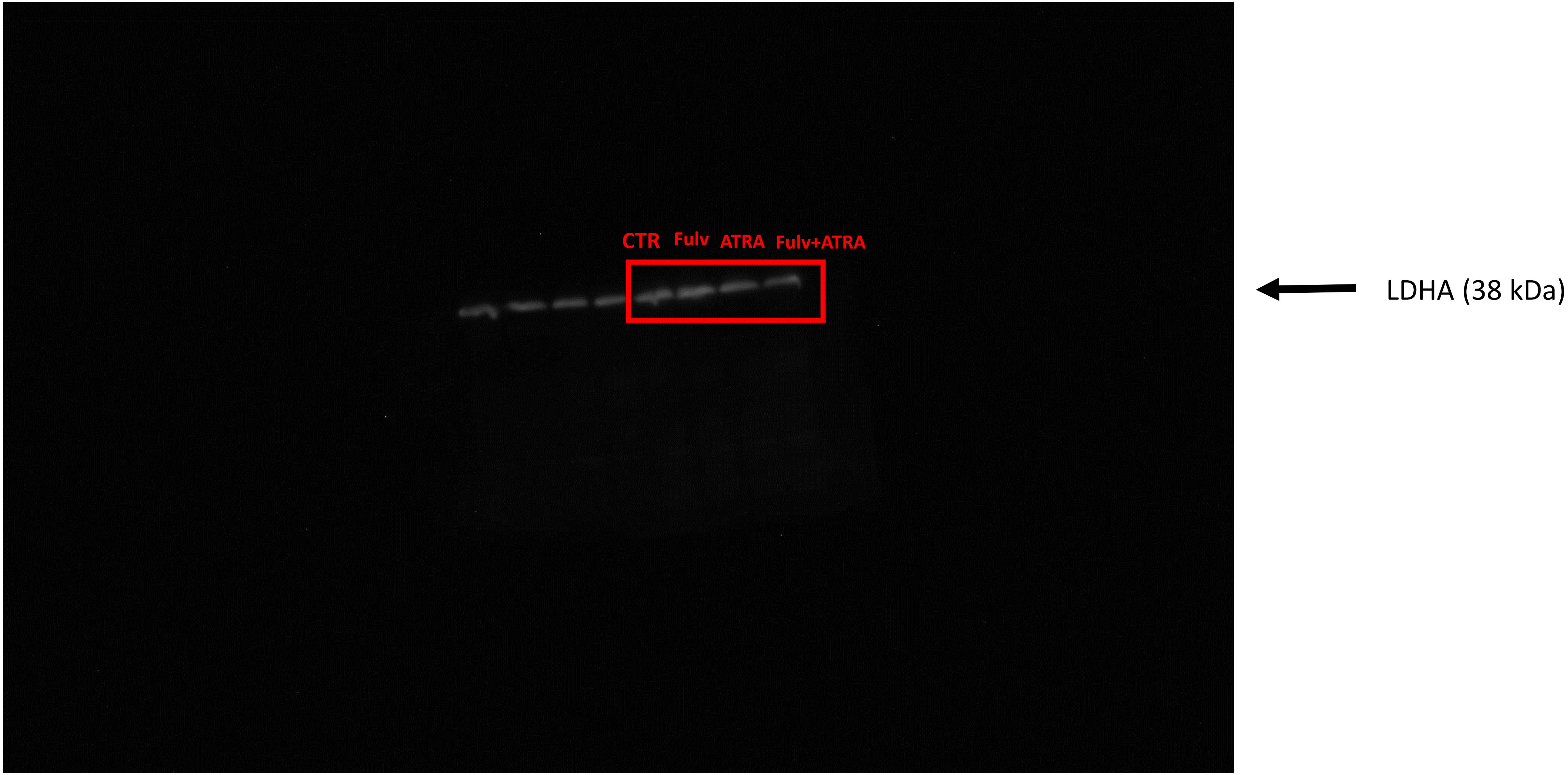

Supplementary fig 5. Effect of ATRA and/or fulvestrant on LDHA glycolytic enzyme in MDA-MB-231 cells. (Full-length blots of figure 6.c)

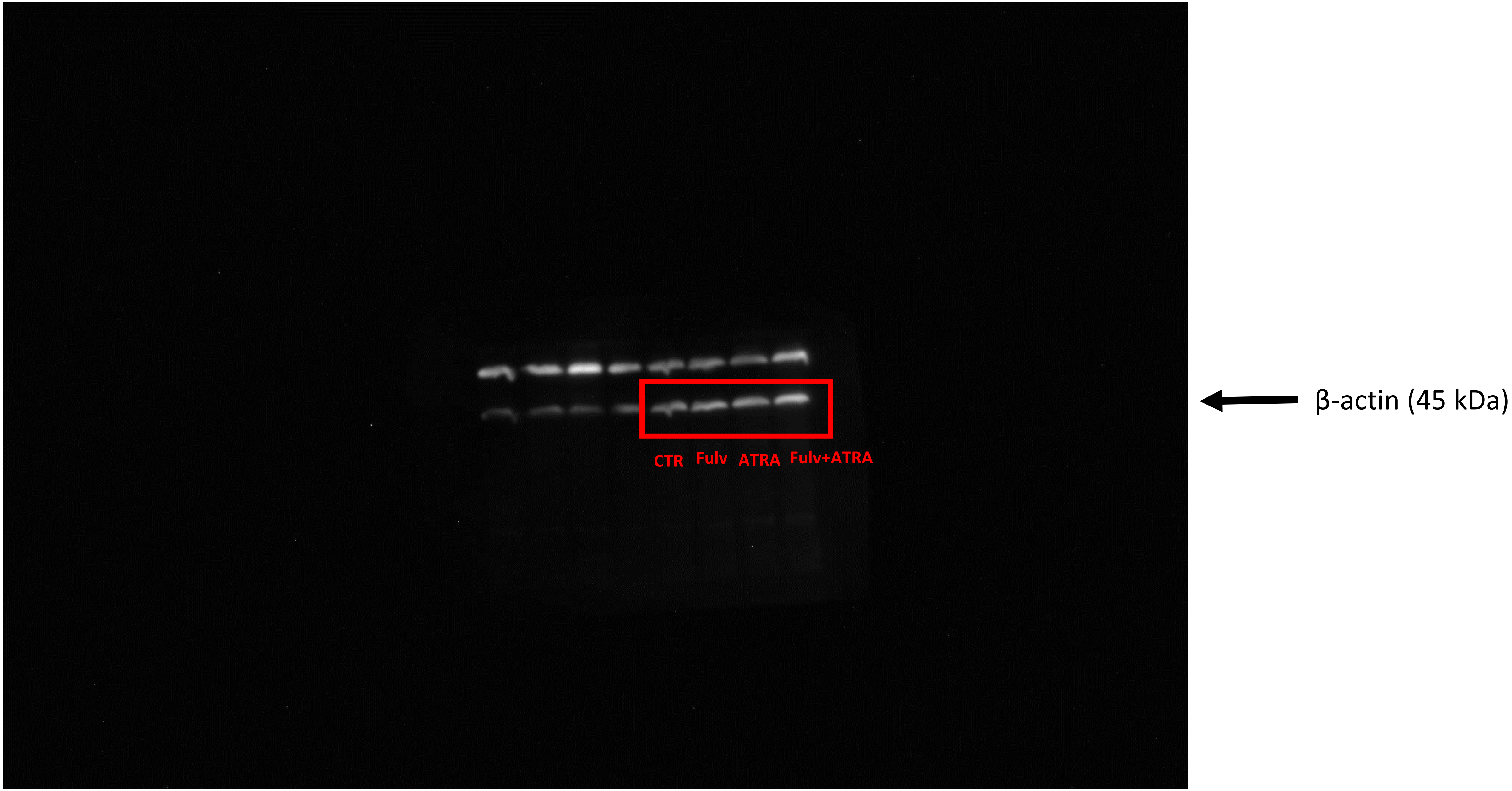

Supplementary fig 5. Effect of ATRA and/or fulvestrant on LDHA glycolytic enzyme in MDA-MB-231 cells. (Full-length blots of figure 6.c)

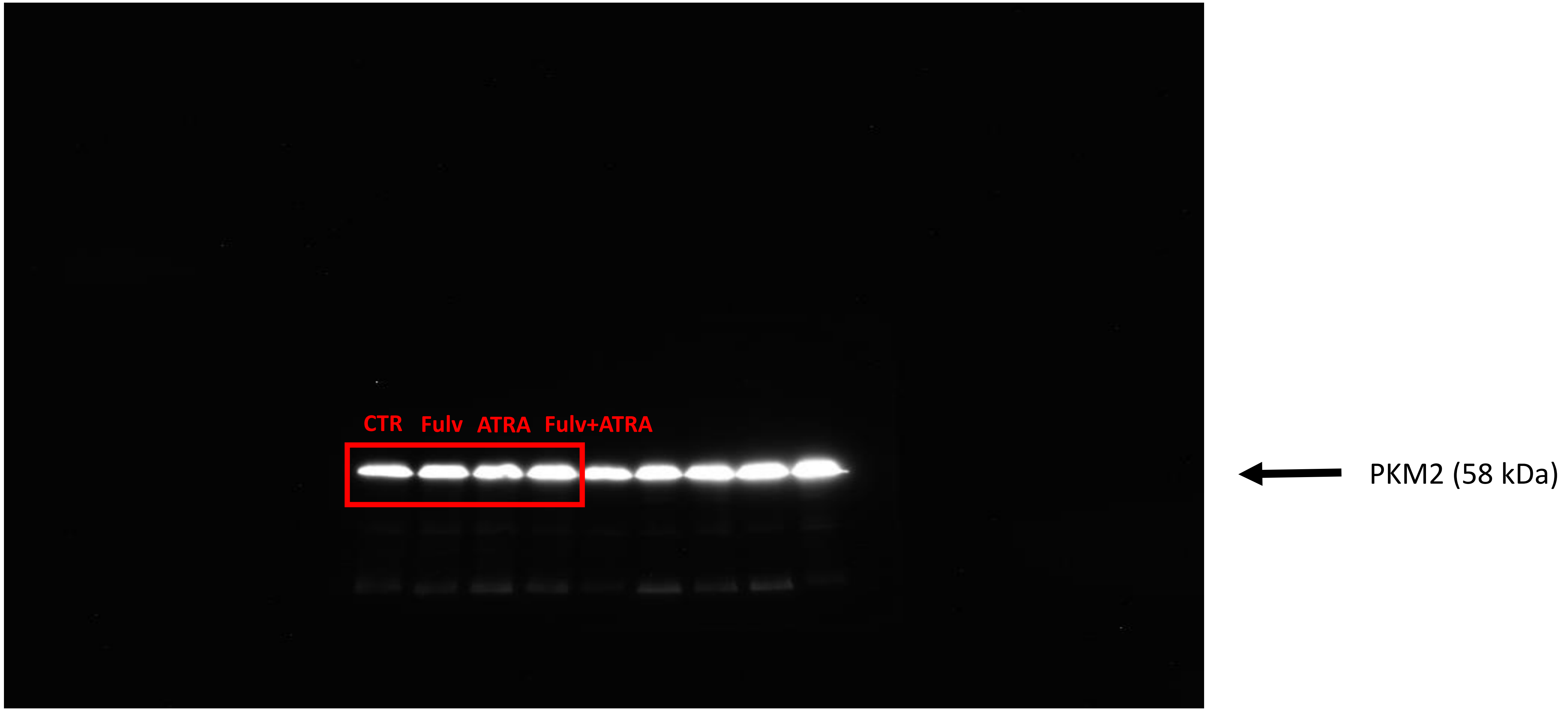

Supplementary fig 6. Effect of ATRA and/or fulvestrant on PKM2 glycolytic enzyme in MDA-MB-231 cells. (Full-length blots of figure 6.e)

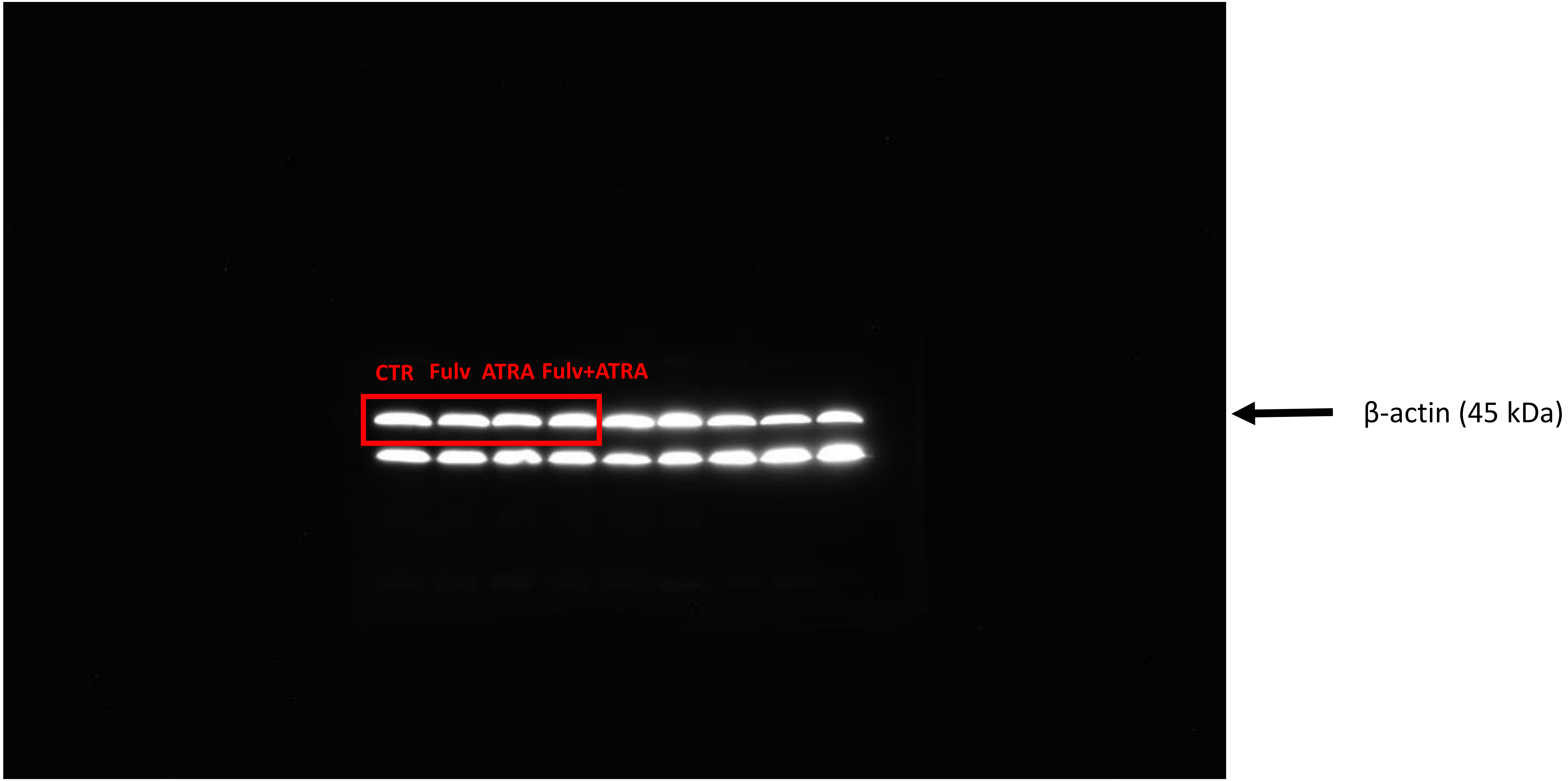

Supplementary fig 6. Effect of ATRA and/or fulvestrant on PKM2 glycolytic enzyme in MDA-MB-231 cells. (Full-length blots of figure 6.e)

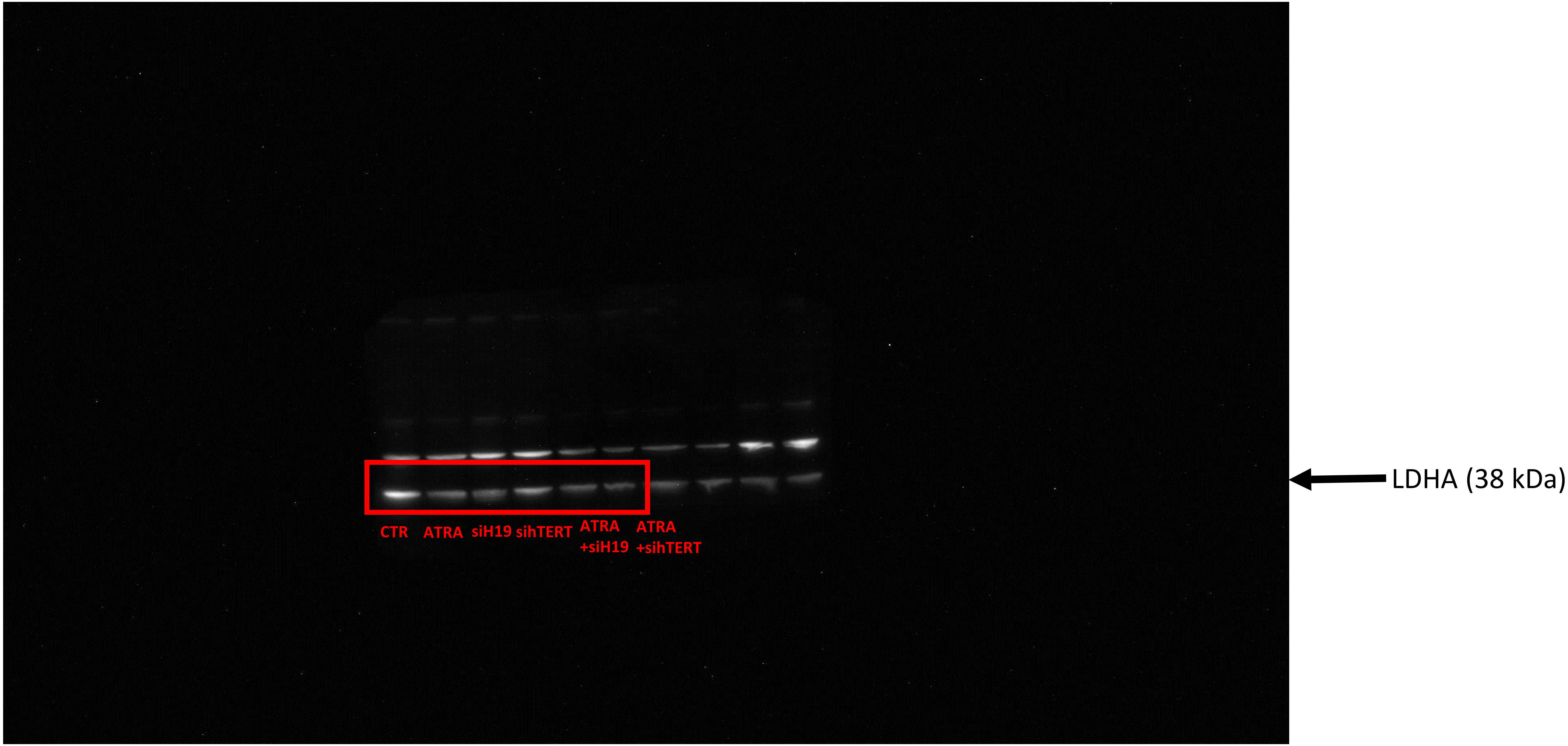

Supplementary fig 7. ATRA reduces LDHA glycolytic enzyme expression through H19 and hTERT in MCF-7 cells. (Full-length blots of fig 8.c)

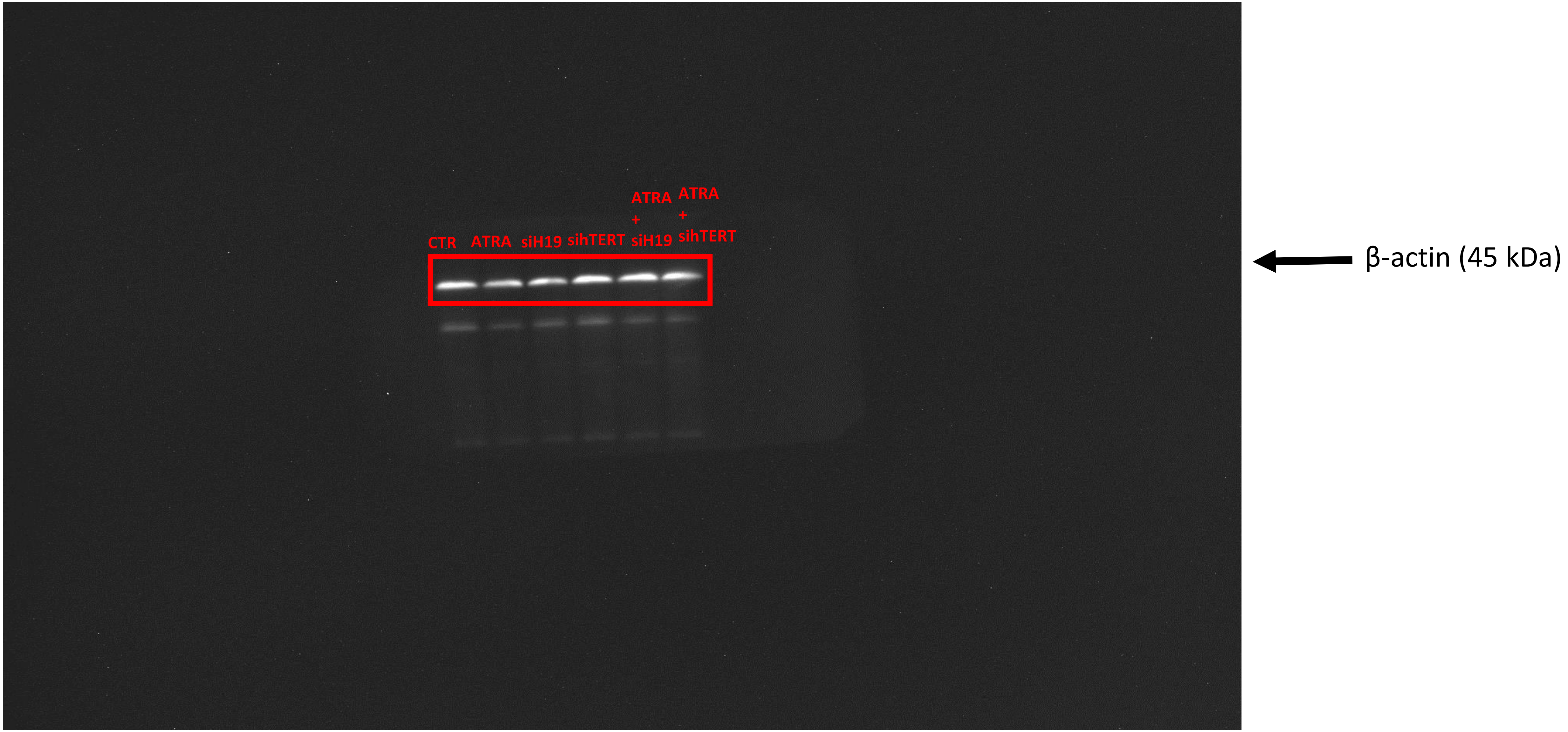

Supplementary fig 7. ATRA reduces LDHA glycolytic enzyme expression through H19 and hTERT in MCF-7 cells. (Full-length blots of fig 8.c)

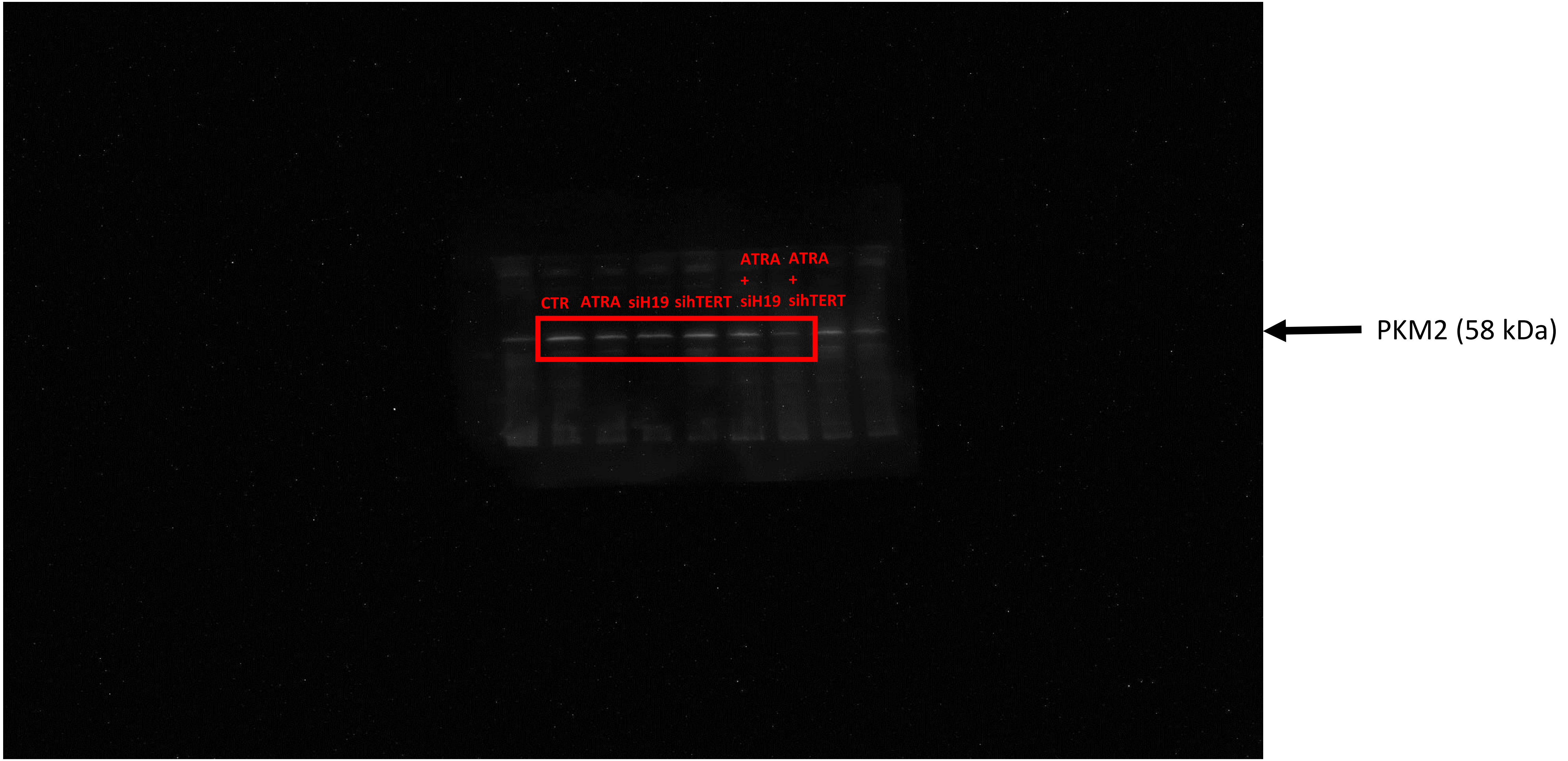

Supplementary fig 8. ATRA reduces PKM2 glycolytic enzyme expression through H19 and hTERT in MCF-7 cells. (Full-length blots of fig 8.e)

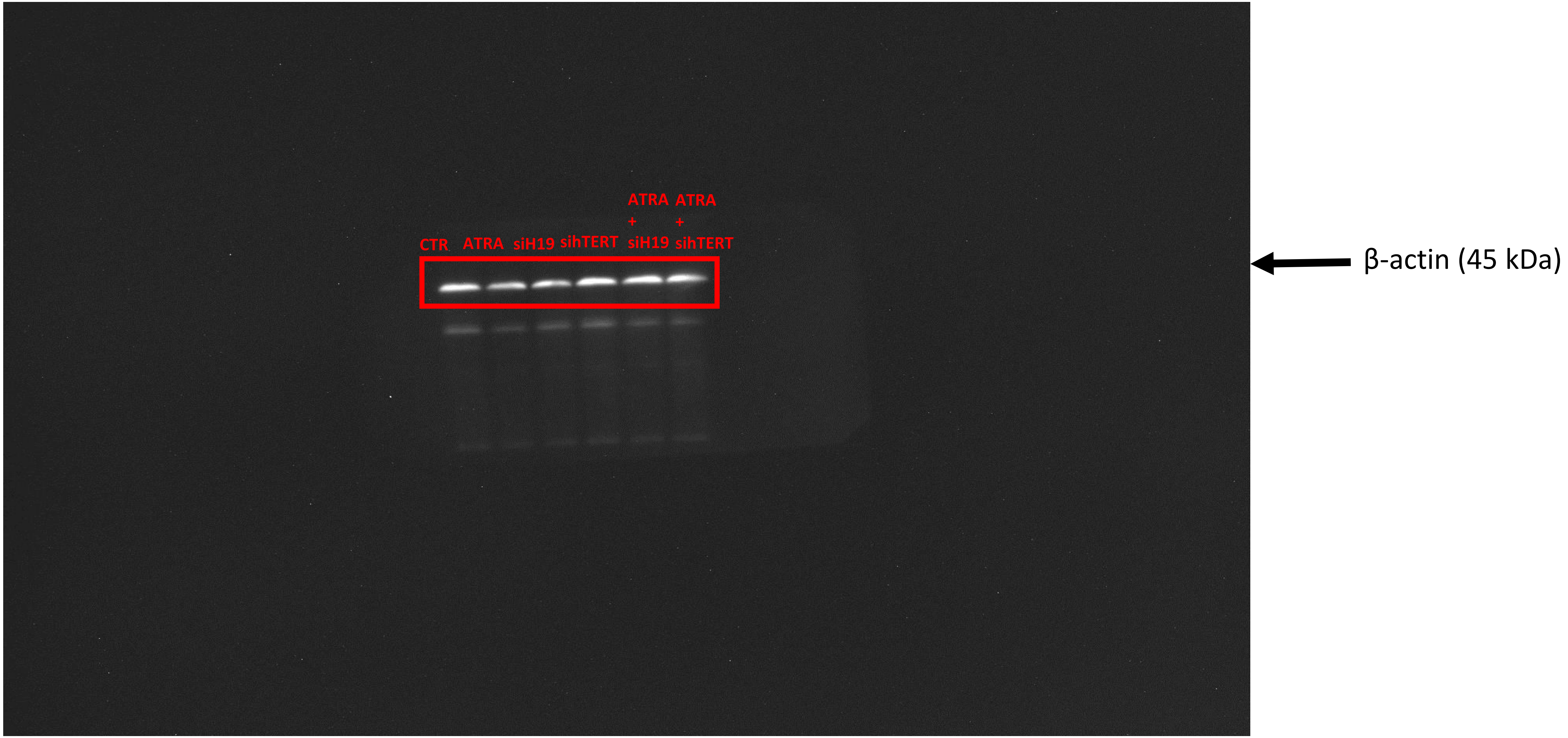

Supplementary fig 8. ATRA reduces PKM2 glycolytic enzyme expression through H19 and hTERT in MCF-7 cells. (Full-length blots of fig 8.e)

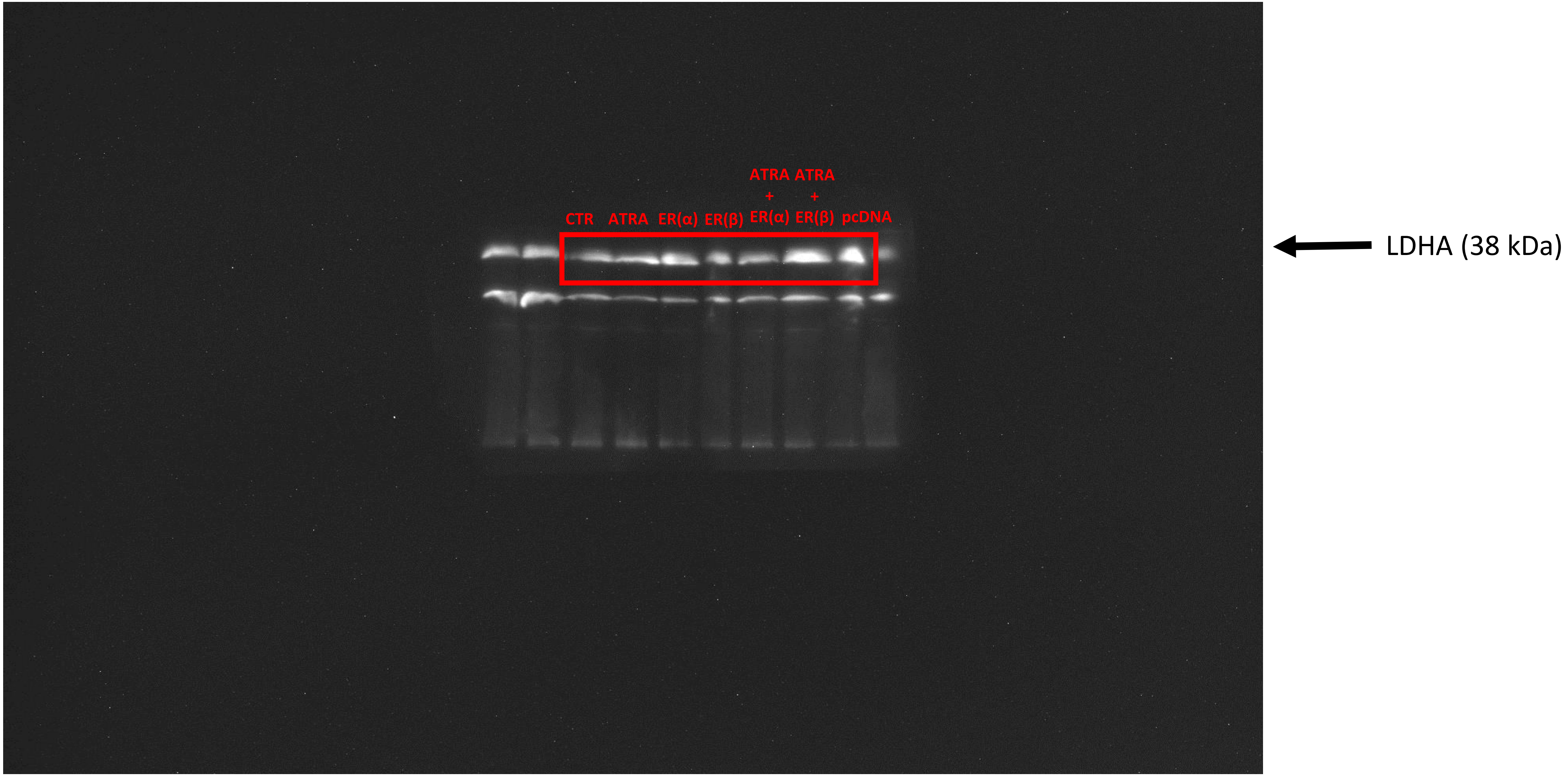

Supplementary fig 9. Upregulated estrogen receptor alpha or beta modulates LDHA in MDA-MB-231 cells. (Full-length blots of fig 10.c)

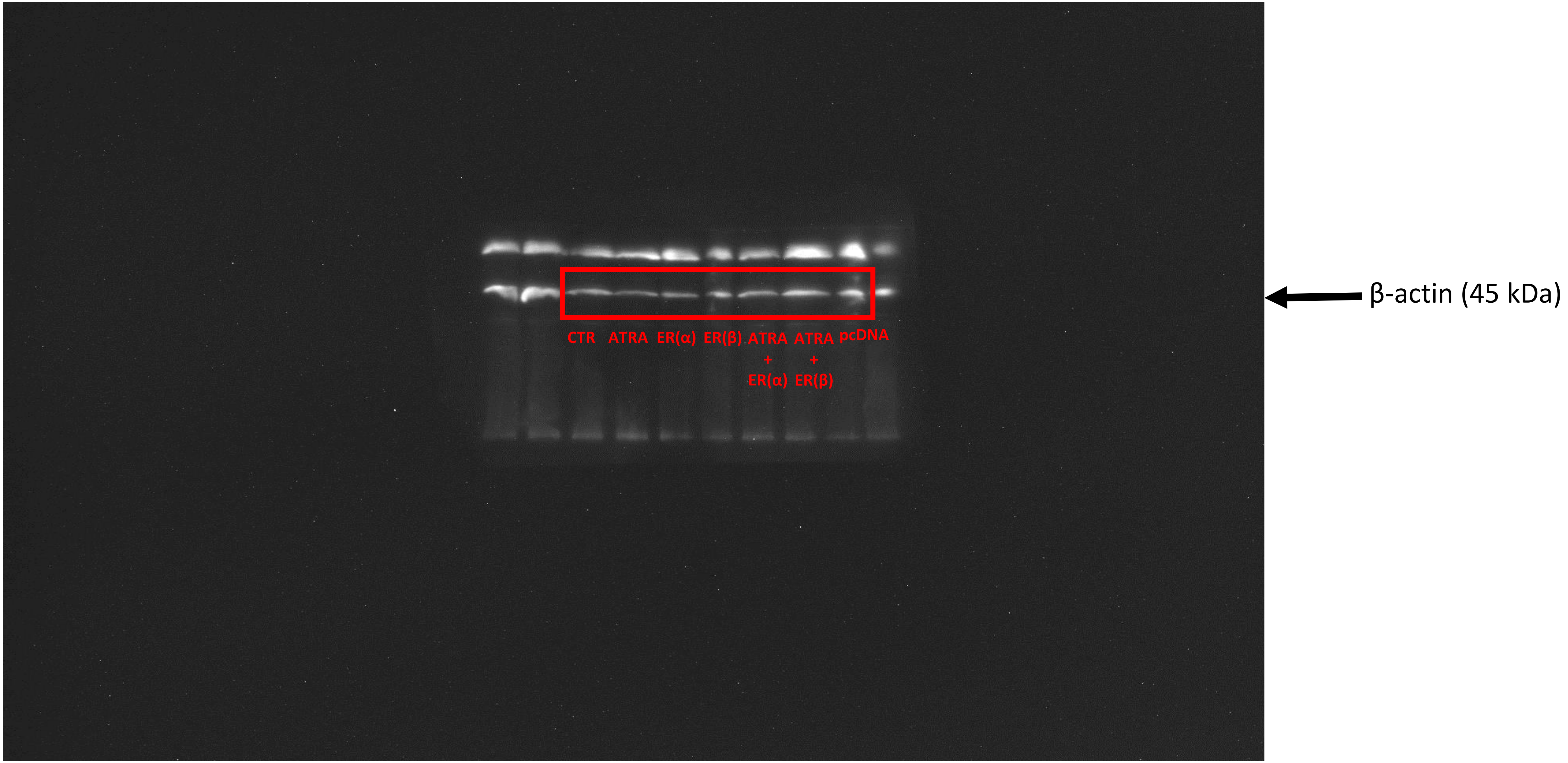

Supplementary fig 9. Upregulated estrogen receptor alpha or beta modulates LDHA in MDA-MB-231 cells. (Full-length blots of fig 10.c)

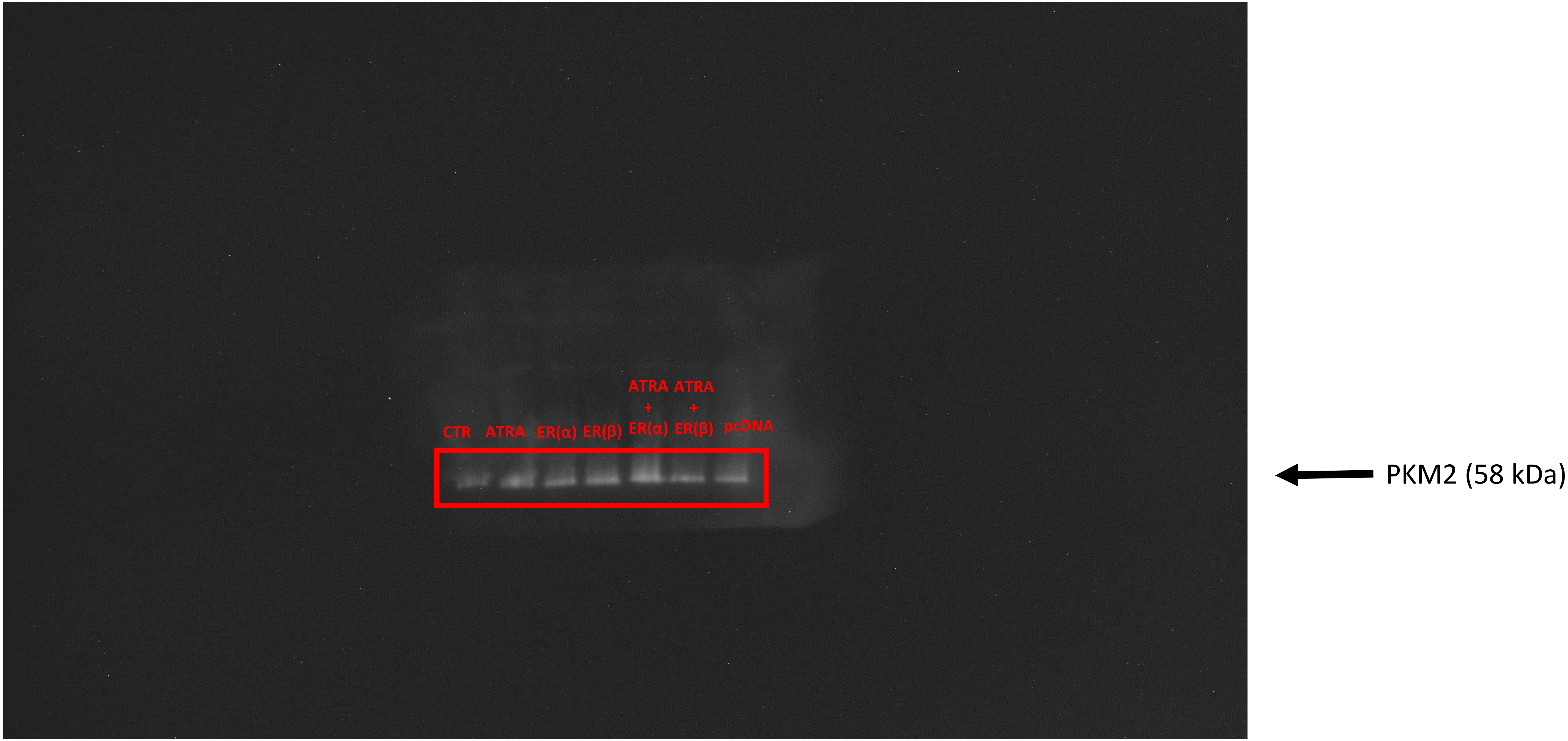

Supplementary fig 10. Upregulated estrogen receptor alpha or beta modulates PKM2 in MDA-MB-231 cells. (Full-length blots of fig 10.e)

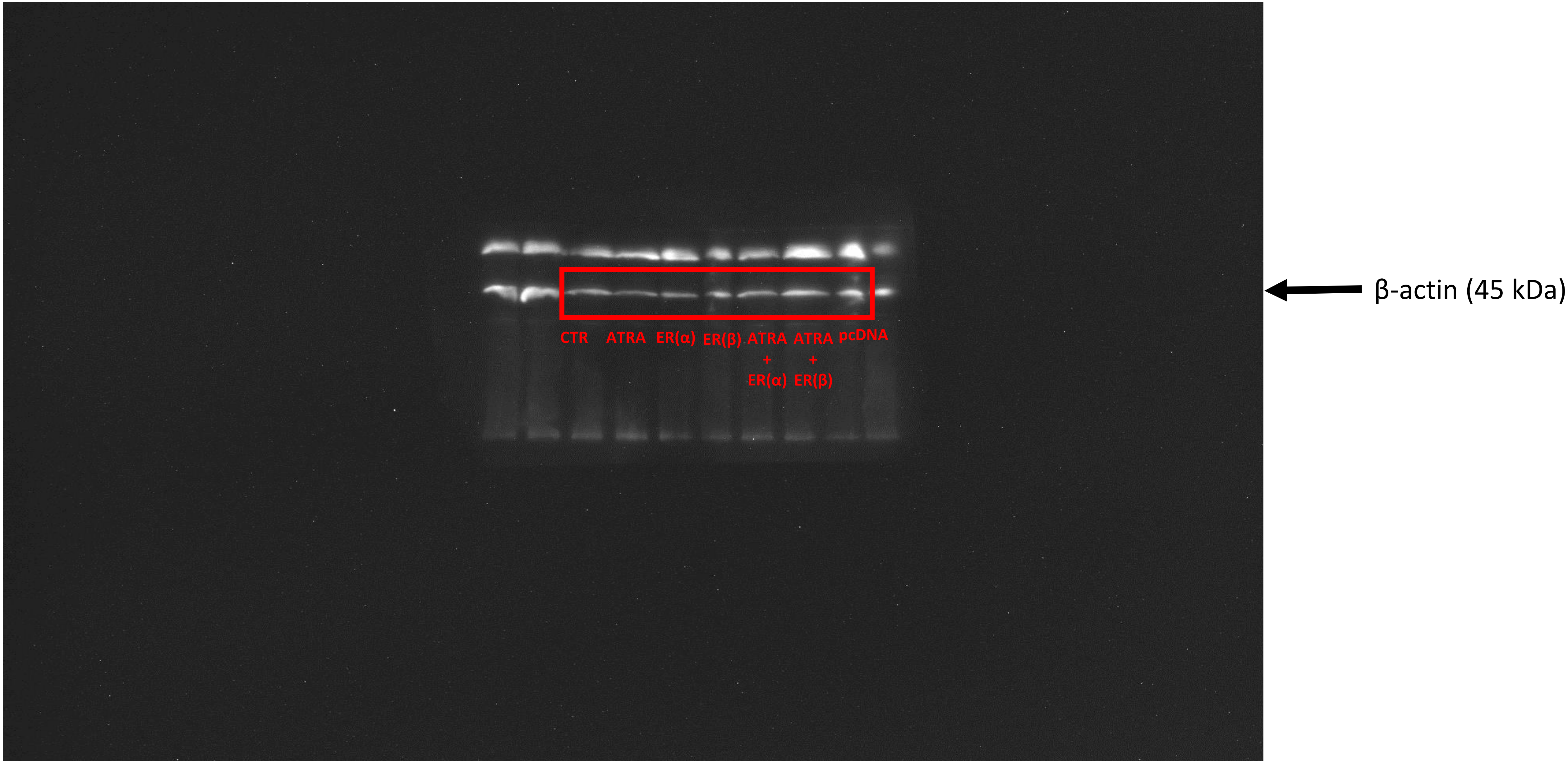

Supplementary fig 10. Upregulated estrogen receptor alpha or beta modulates PKM2 in MDA-MB-231 cells. (Full-length blots of fig 10.e)
